# PPARγ activation but not PPARγ haplodeficiency affects proangiogenic potential of endothelial cells and bone marrow-derived progenitors

**DOI:** 10.1186/s12933-014-0150-7

**Published:** 2014-11-01

**Authors:** Jerzy Kotlinowski, Anna Grochot-Przeczek, Hevidar Taha, Magdalena Kozakowska, Bartosz Pilecki, Klaudia Skrzypek, Aleksandra Bartelik, Rafal Derlacz, Anton J G Horrevoets, Attila Pap, Laszlo Nagy, Jozef Dulak, Alicja Jozkowicz

**Affiliations:** Department of Medical Biotechnology, Krakow, Poland; Department of Biophysics, Faculty of Biochemistry, Biophysics and Biotechnology, Jagiellonian University, Krakow, Poland; R & D Department, Adamed Ltd, Pienkow, Poland; Department of Metabolic Regulation, Institute of Biochemistry, Faculty of Biology, University of Warsaw, Warsaw, Poland; Department of Molecular Cell Biology and Immunology, VU University Medical Center, Amsterdam, The Netherlands; Department of Biochemistry and Molecular Biology, Faculty of Medicine, University of Debrecen, Debrecen, Hungary; MTA-DE “Lendület” Immunogenomics Research Group, University of Debrecen, Debrecen, Hungary; Sanford-Burnham Medical Research Institute, Orlando, FL USA; Department of Molecular Neuropharmacology, Institute of Pharmacology Polish Academy of Science, Krakow, Poland

**Keywords:** Diabetes, PPARγ, Therapeutic angiogenesis, Endothelial progenitor cells

## Abstract

**Background:**

Peroxisome proliferator-activated receptor-γ (PPARγ) agonists, which have been used as insulin sensitizers in diabetic patients, may improve functions of endothelial cells (ECs). We investigated the effect of PPARγ on angiogenic activities of murine ECs and bone marrow-derived proangiogenic cells (PACs).

**Methods:**

PACs were isolated from bone marrow of 10–12 weeks old, wild type, db/db and PPARγ heterozygous animals. Cells were cultured on fibronectin and gelatin coated dishes in EGM-2MV medium. For *in vitro* stimulations, rosiglitazone (10 μmol/L) or GW9662 (10 μmol/L) were added to 80% confluent cell cultures for 24 hours. Angiogenic potential of PACs and ECs was tested *in vitro* and *in vivo* in wound healing assay and hind limb ischemia model.

**Results:**

ECs and PACs isolated from diabetic db/db mice displayed a reduced angiogenic potential in *ex vivo* and *in vitro* assays, the effect partially rescued by incubation of cells with rosiglitazone (PPARγ activator). Correction of diabetes by administration of rosiglitazone *in vivo* did not improve angiogenic potential of isolated PACs or ECs. In a hind limb ischemia model we demonstrated that local injection of conditioned media harvested from wild type PACs improved the blood flow restoration in db/db mice, confirming the importance of paracrine action of the bone marrow-derived cells.

Transcriptome analysis showed an upregulation of prooxidative and proinflammatory pathways, and downregulation of several proangiogenic genes in db/db PACs. Interestingly, db/db PACs had also a decreased level of PPARγ and changed expression of PPARγ-regulated genes. Using normoglycemic PPARγ^+/−^ mice we demonstrated that reduced expression of PPARγ does not influence neovascularization either in wound healing or in hind limb ischemia models.

**Conclusions:**

In summary, activation of PPARγ by rosiglitazone improves angiogenic potential of diabetic ECs and PACs, but decreased expression of PPARγ in diabetes does not impair angiogenesis.

**Electronic supplementary material:**

The online version of this article (doi:10.1186/s12933-014-0150-7) contains supplementary material, which is available to authorized users.

## Introduction

Peroxisome proliferator-activated receptor gamma (PPARγ) is a ligand-dependent transcription factor of nuclear receptor superfamily, involved in regulation of lipid metabolism, insulin sensitivity, and inflammatory response [1,2]. Synthetic PPARγ agonists (thiazolidinediones), such as rosiglitazone (ROSI), pioglitazone, and troglitazone, have been used for many years in patients with type 2 diabetes (T2DM) to improve insulin resistance and ameliorate hyperglycemia [3].

Diabetes, due to hyperglycemia and insulin resistance, is associated with poor outcomes due to endothelial dysfunction and impaired angiogenesis, followed by vascular occlusive events, decreased vascular density in ischemic tissues, and delayed wound healing [4,5]. Proper vascular repair and angiogenesis depend both on mature endothelial cells and endothelial progenitors [6,7]. Bone marrow-derived endothelial progenitor cells (EPCs) were first characterized in 1997 as a CD34^+^ population mobilized into the peripheral blood in response to ischemia, capable of incorporating into injured vessels and involved in tissue revascularization [8]. In further studies a broad spectrum of protocols for isolation or characterization of EPCs has been used, which makes the comparison of the results difficult. It seems, however, that cells named EPCs are rather a heterogenous population containing mainly monocytes with angiogenic properties [9]. They can produce a range of growth factors including the vascular endothelial growth factor-A (VEGF-A), stromal cell-derived factor-1 (SDF-1), or interleukin-8 (IL-8), and this paracrine activity is supposed to be the most important for compensatory vascularization in ischemic tissues [10-12]. Despite lack of standardized definition, the analyzes have consistently indicated that the bone marrow-derived proangiogenic cells are altered in type 2 diabetes. Either in animal models or in human patients, diabetes reduces number of EPCs, impairs their mobilization, and decreases their angiogenic potential [13-18].

The deleterious effects of diabetes on mature endothelium and bone marrow-derived proangiogenic cells can be favorably modulated by PPARγ agonists. Hence, treatment with rosiglitazone facilitated reendothelialization in diabetic patients, through decreased ROS generation and improved bioavailability of nitric oxide (NO) in endothelial cells [19]. It also increased the number of circulating EPCs and normalized their migratory activity [20]. Importantly, PPARγ agonists may improve endothelial functions and angiogenic capacities independently of insulin sensitizing in normoglycemic patients [21-23]. They were also shown to upregulate expression of VEGF in rat and human vascular smooth muscle cells [24,25].

Direct proangiogenic activity of PPARγ agonists have been described in human umbilical vein endothelial cells (HUVECs), where expression of VEGF receptor-2 (VEGFR-2) and formation of cord-like structures on Matrigel were enhanced in response to troglitazone [26]. However, the effect of PPARγ on angiogenic activities of endothelial cells is still not clear, as inhibition of cord-like structure formation, decreased proliferation, and impaired migration have also been reported in HUVECs treated with PPARγ agonists [27,28]. Moreover, while there are numerous papers on vascular effects of PPARγ agonist, much less is known about potential consequences of PPARγ deficiency or inhibition. It has been shown that administration of PPARγ antagonist (T0070907) in pregnant rats led to endothelial dysfunction, reduced expression of VEGF and increased level of plasma soluble VEGF receptor-1 (sVEGFR-1), which acts as a VEGF scavenger [29]. Accordingly, lack of PPARγ impaired vascular proliferation and led to vasculature defects in murine placentas, what was a unique cause of PPARγ^−/−^ lethality [30]. These observations indicate that proper expression of PPARγ may be an important issue in regulation of angiogenesis. Therefore, we examined the effects of PPARγ activation and PPARγ inhibition on angiogenic activities of endothelial and bone marrow-derived proangiogenic cells (PACs) in diabetic and normoglycemic conditions.

## Materials and methods

### Reagents

Endothelial cell basal medium-2 (EBM-2) with a growth factor supplement was obtained from Lonza. MCDB-131 medium, phosphate buffered saline (PBS), Accutase and fetal bovine serum (FBS) were from PAA Laboratories. BS1 lectin was purchased from Vector Laboratories. DiI labeled acetylated low density lipoproteins (DiI-AcLDL) were from Invitrogen and QCM cell migration assay from Chemicon. Mouse VEGF ELISA, mouse SDF-1 ELISA, and growth factor-reduced Matrigel were obtained from R&D Systems. Fenozol was obtained from A&A Biotechnology. Anti-mouse proliferating cell nuclear antigen (PCNA) antibody was purchased from Dako. Anti-mouse antibodies against stem cell antigen-1 (Sca-1), chemokine receptor type-4 (CXCR4), CD45, and kinase insert domain receptor (KDR, VEGFR-2) used for immunophenotyping of PACs, antibodies recognizing mouse CD31 used for immunohistochemistry and PharmLyse were obtained from BD Biosciences. All other reagents were purchased from Sigma.

### Cell culture

PACs were cultured as described earlier [12]. In short: total bone marrow cells were flashed from tibias and femurs of mice and centrifuged on Ficoll gradient. Cells from the interphase were washed three times with PBS, seeded on fibronectin (20 μg/mL) and gelatin (0.25%) coated dishes in EGM-2MV medium (EBM-2 with growth factor supplement) containing 10% FBS, and cultured up to 80% confluence for 9–11 days in standard conditions (humidified atmosphere, 21% O_2_, 5% CO_2_). In some experiments the cells were cultured in hypoxia (2% O_2_, 5% CO_2_ for 24 hours). Phenotype of the cultured cells was validated by uptake of DiI-AcLDL (10 μg/mL), binding of FITC-labeled BS1 lectin, expression of progenitor, endothelial or hematopoietic marker mRNAs, and presence of Sca-1, CXCR4, KDR and CD45 proteins.

For *in vitro* stimulations, rosiglitazone (10 μmol/L) or GW9662 (10 μmol/L) were added to 80% confluent cell cultures for 24 hours. In case of PPARγ inhibition with GW9662 followed by stimulation with rosiglitazone the inhibitor was added first, 30 minutes ahead.

HUVECs were cultured in MCDB-131 complete medium, supplemented with 10% FBS endothelial cell growth supplement (ECGS) and hydrocortizone [12].

### Animals

All experiments were approved by the Local Ethical Committee for Animal Research at the Jagiellonian University. Mice were handled according to good animal practice in science, with a food and water access *ad libitum*. Wild type and db/db mice (C57BLKS background) were purchased from Taconic (Denmark), whereas PPARγ wild type and heterozygous animals (C57BL/6 J background) were kindly provided by Dr. Laszlo Nagy (University of Debrecen). In all experiments 10–12 weeks old mice were employed. For *in vivo* drug delivery mice were treated daily for two weeks by oral gavage either with rosiglitazone (10 mg/kg body weight) or placebo (control WT and db/db mice).

### Migration

80% confluent PACs were detached using Accutase. Next, 10,000 cells were seeded in EBM-2 empty medium on the top of 8-μm transwell filters and stimulated with rosiglitazone (10 μmol/L) and/or GW9662 (10 μmol/L, added 30 minutes before rosiglitazone). Lower chamber was filled with EGM-2MV medium supplemented with 10% FBS. Cells were incubated overnight under standard culture conditions. Then, the migrated cells on the underside of the membrane were fixed in 3% paraformaldehyde for 10 minutes, washed with PBS and stained with crystal violet solution, according to vendor's protocol. For each sample the number of cells was calculated as mean cell count of 10 randomly-selected microscopic fields using Nikon Eclipse TX-100 microscope.

### Tube formation on matrigel

Growth factor-reduced Matrigel was poured into a 96-well plate (50 μL/well) and incubated at 37°C for 15 minutes. Then 20,000 PACs were seeded to each well and stimulated with rosiglitazone (10 μmol/L) and/or GW9662 (10 μmol/L, added 30 minutes before rosiglitazone). Resulting tube-like structures were counted in whole well after the 16 h incubation period using Nikon Eclipse TX-100 microscope.

### Proliferation assay

PACs were seeded in chamber slides and cultured in standard conditions until reaching a confluence of 70%. Proliferating cells were stained with anti-mouse PCNA antibody and PCNA-positive cells were counted using the fluorescence microscope (Nikon Eclipse TX-100).

### Flow cytometry

PACs number in the peripheral blood and in the bone marrow was measured on the basis of analysis of CD45^−^KDR^+^Sca-1^+^ population. Peripheral blood was harvested from *vena cava superior* into heparinized syringe, whereas bone marrow was flushed from tibias and femurs. Next, red blood cells were removed with PharmLyse buffer and, after washing, cells were incubated with anti-mouse antibodies (APC-Cy7 CD45, FITC Sca-1 and APC KDR) for 30 minutes at 4°C in RPMI 1640 medium containing 2% FBS. Data were collected from at least 1,000,000 events using a cytofluorometer (LSR II; Becton Dickinson) and analyzed using FACSDiva software (BD Biosciences).

### ELISA

Concentrations of VEGF and SDF-1 proteins in blood plasma and tissue lysates were measured by ELISA tests according to vendor's protocol.

### Gene expression analysis

Total RNA was isolated from PACs and from bone marrow (after lysis of red blood cells) with a modified guanidinium isothiocyanate method. For cDNA synthesis 0.5 μg RNA was used. Gene expression was measured by real time PCR (StepOnePlus, Applied Biosystems) according to the protocol: 95°C for 5 minutes followed by 40 cycles of melting at 95°C – 30 s, annealing at 58-62°C – 60 s, elongation at 72°C – 45 s. Primer sequences, annealing temperatures, and length of PCR products are listed in Additional file 1: Table S1.

### Wound healing

Wound healing assay was performed as described previously [31]. Two full-thickness circular wounds (4 mm in diameter) on each animal were created using disposable biopsy punch. Each wound was photographed every day and analyzed using ImageJ software.

### Hind limb ischemia

Left femoral artery ligation was performed to induce limb ischemia in WT, PPARγ deficient and db/db mice. The superficial blood flow of the ischemic and contralateral foot was analyzed by a laser Doppler flowmeter (Periflux). Ratio between blood flow in the ischemic foot and in contralateral foot was calculated and used as an index of blood flow recovery.

For cell therapy, PACs were isolated from WT mice and cultured up to confluence in standard conditions. Then the growth medium was changed to EBM-2 containing 0.5% FBS, which was harvested after a 24 h incubation period to obtain PAC-conditioned medium. Conditioned media were frozen for further use. Diabetic mice (db/db) were subjected to femoral artery ligation and next day the conditioned media, control media (EBM-2 with 0.5% FBS) or PACs (250,000 cells in control medium) in a total volume of 50 μL were injected intramuscularly into 3 sites of ischemic gastrocnemius muscle. Blood flow measurements were performed weekly till 28^th^ day after injections. At the end of experiment gastrocnemius muscles were frozen for immunohistochemical and histochemical analyzes.

### Immunohistochemical staining

Vascularization of gastrocnemius muscles was assessed by CD31 staining of endothelial cells. For this purpose, frozen sections of muscle (6 μm) were dried for 1 h in room temperature and then fixed for 10 minutes in acetone (4°C). Sections were blocked in 10% goat serum, 0.05% Tween-20, 0.1% TritonX-100 for 1 h in room temperature, and then incubated with anti-mouse CD31 antibodies for 1.5 h. After washing, sections were incubated with rhodamine-labeled secondary antibodies for 30 minutes in room temperature, washed and mounted in fluorescent mounting medium. The number of capillaries per muscle fiber were counted from 10 random fields for each specimen using Nikon Eclipse TX-100 microscope.

### Statistical analysis

Results are expressed as mean ± SEM. *In vitro* experiments were carried out 3–10 times. *In vivo* experiments were done with 5–9 mice per group. Two tailed Student’s *t* test was used for comparison of two groups, while one-way ANOVA with Bonferroni posttest way applied for comparison of multiple groups.

Full length description of methods is available in the supplemental file.

## Results

### Characterization of bone marrow-derived PACs

Bone marrow-derived proangiogenic cells isolated from healthy C57BL6/J (wild type, WT) mice and cultured for 9–11 days under conditions promoting the endothelial differentiation, generated a heterogeneous population, with 80-90% of cells incorporating acLDL and binding BS1 lectin, capable of forming the capillary-like structures after seeding on Matrigel (Additional file 2: Figure S1A-D), which confirmed our earlier observation [12]. Such population was 450- to 650-fold enriched in CD45^−^KDR^+^Sca-1^+^ cells comparing to freshly isolated bone marrow, although their content, even after such enrichment, did not exceed 0.5%. PACs derived from db/db diabetic mice displayed similar morphology or acLDL and BS1 staining and similar level of enrichment in CD45^−^KDR^+^Sca-1^+^ cells (data not shown).

Transcriptome analysis revealed that expression of genes typical for progenitor cells (e.g. Sca-1, c-kit, CD34), endothelial cells (e.g. KC, vascular cell adhesion molecule-1 (VCAM-1), endoglin, von Willebrand factor (vWF), and hematopoietic cells (e.g. CD11b, CD14) can be detected in the heterogeneous PAC populations at the levels similar in WT and db/db cells (data not shown). FACS immunophenotyping showed that vast majority of PACs expressed CD45, a pan-hematopoietic marker, and proportion of such cells was slightly higher in the db/db than in WT cultures (85 ± 1% *vs*. 77 ± 1%). On the other hand, frequency of KDR-positive cells, these expressing typical endothelial marker, was lower in population isolated from db/db mice (5 ± 1% *vs*.10 ± 2%. More than 60% of both WT and db/db cells were positive for CXCR4 and Sca-1 markers, preferentially expressed on progenitor cells (Additional file 2: Figure S1E).

### Angiogenic potential of PACs

**Figure 1:**
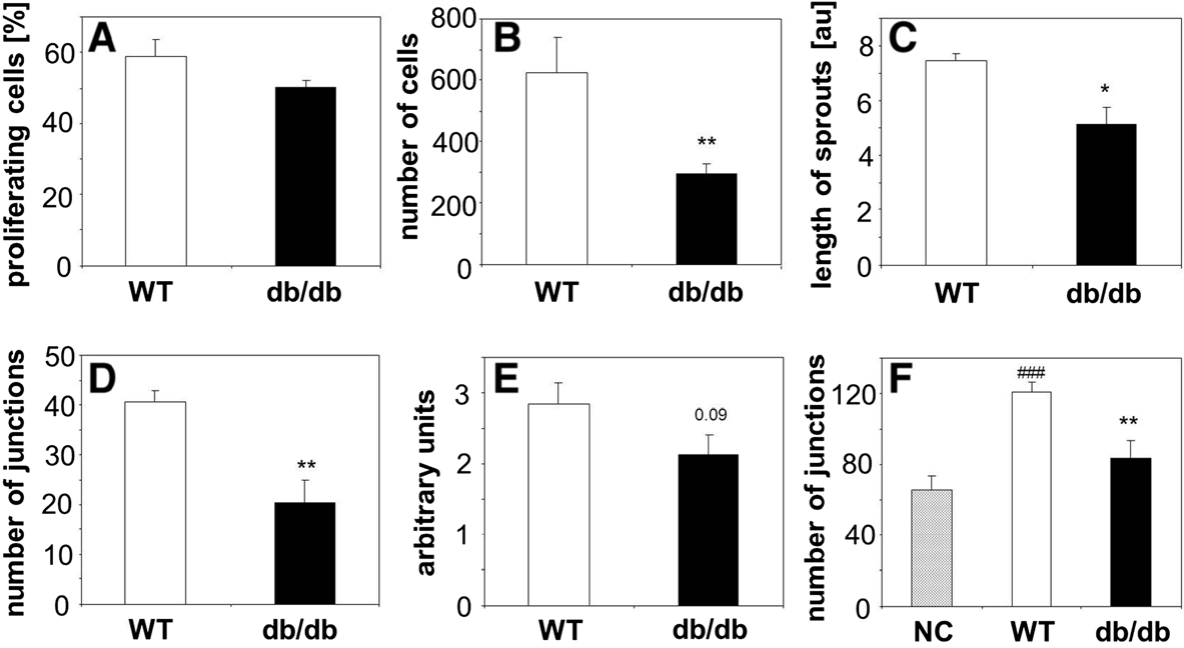
**Angiogenic potential of PACs and endothelial cells from wild type (WT) and diabetic (db/db) mice. A**: Proliferation of PACs. Immunohistochemical detection of PCNA-positive cells. **B**: Migration of PACs in modified Boyden chambers. **C**: Formation of sprouts from PACs spheroids embedded in collagen gel, shown as a total length of sprouts per one spheroid. **D**: Formation of cords by PACs seeded on Matrigel shown as a number of cord junctions per microscopic field. **E**: Formation of capillaries by endothelial cells from aortic rings isolated from WT or db/db mice and plated on Matrigel. **F**: Formation of cords by HUVECs seeded on Matrigel and stimulated with empty medium (NC, negative control) or conditioned media from WT PACs and db/db PACs, shown as a number of cord junctions per microscopic field. Each bar represents mean + SEM. N = 4-5 (1A-E), N = 8-9 (1F), ^*^ *p* < 0.05, ^**^ *p* < 0.01 *versus* WT, ^###^ *p* < 0.001 *versus* NC.

In a set of *in vitro* experiments we compared angiogenic potential of wild type and db/db PACs. Spontaneous proliferation, measured using immunohistochemical detection of PCNA, was similar in cells isolated from healthy and diabetic animals (Figure 1A). All other activities measured, namely migration (Figure 1B), sprouting of capillaries from PACs spheroids embedded in collagen (Figure 1C), and formation of cord-like structures on Matrigel (Figure 1D) were attenuated in db/db cells, indicating their impaired angiogenic potential. Similarly, angiogenic activity of mature endothelial cells derived from aorta of db/db mice tended to be weaker, as illustrated by the results of semiquantitative ring assay (Figure 1E).

Since angiogenesis can be stimulated by bone marrow-derived cells in a paracrine way, we also evaluated the effect of conditioned media collected from the cultured PACs on HUVEC cells seeded on Matrigel. As shown in Figure 1F, PACs isolated from diabetic mice displayed a weaker paracrine activity – they induced formation of sparser capillary network by HUVEC endothelial cells (Figure 1F).

### Effect of rosiglitazone on angiogenic potential of PACs

There are discrepancies between papers describing either proangiogenic [5,20-22,32], antiangiogenic [27,28,33-35] or biphasic [36] effects of PPARγ agonists. Therefore we checked whether impaired angiogenic potential of PACs isolated from db/db mice can be corrected by rosiglitazone. Cells were exposed to rosiglitazone (10 μmol/L) for 24 h. This resulted in a significant upregulation of PPAR activity, which we tested using PPRE-luciferase reporter plasmid (data not shown). To confirm PPARγ-specificity, some cells were preincubated with GW9662 (10 μmol/L, 30 minutes), PPARγ antagonist.

**Figure 2:**
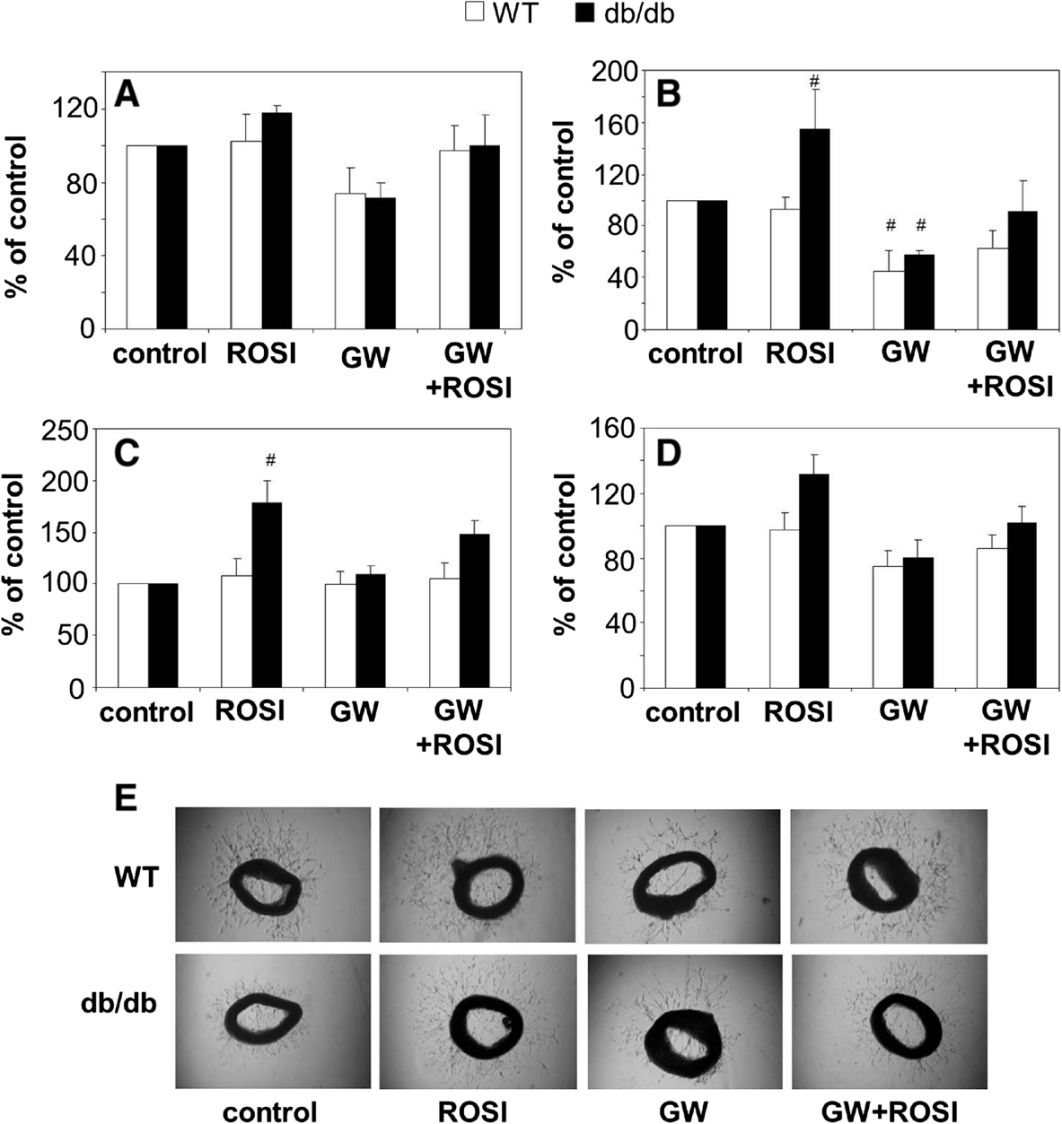
**Effect of PPARγ agonist rosiglitazone (ROSI, 10 μmol/L, 24 h) and PPARγ antagonist GW9662 (GW, 10 μmol/L, 24 h) on angiogenic potential of PACs and endothelial cells from wild type (WT) or diabetic (db/db) mice. A**: Proliferation of PACs. Immunohistochemical detection of PCNA-positive cells. **B**: Migration of PACs in modified Boyden chambers. **C**: Formation of cords by PACs seeded on Matrigel, shown as a number of cord junctions per microscopic field. **D**: Formation of capillaries by endothelial cells from aortic rings isolated from WT or db/db mice and embedded in Matrigel. **E**: representative pictures showing aortic rings. Each bar represents mean + SEM, expressed as percentage of values for untreated control. N = 4-5, ^#^ *p* < 0.05 *versus* control.

Modulation of PPARγ activity did not influence significantly the proliferation of PACs (Figure 2A), although there was a tendency toward inhibition of proliferation in cells treated with GW9662 (*p* = 0.08 and *p* = 0.07 in WT and db/db cells). In contrast, migration (Figure 2B) and formation of cord-like structures on Matrigel (Figure 2C), two activities which were impaired in PACs isolated from db/db mice, were rescued in db/db cells incubated with rosiglitazone. This effect was at least partially blocked by GW9662, confirming the PPARγ-dependency. Interestingly, rosiglitazone did not improve migration or formation of cord-like structures in wild type PACs (Figure 2B,C). Also, rosiglitazone showed a tendency to augment angiogenic activity of mature endothelial cells (*p* = 0.08) in aortal rings derived from db/db mice, without effects on the cells isolated from wild type animals (Figure 2D,E).

**Figure 3:**
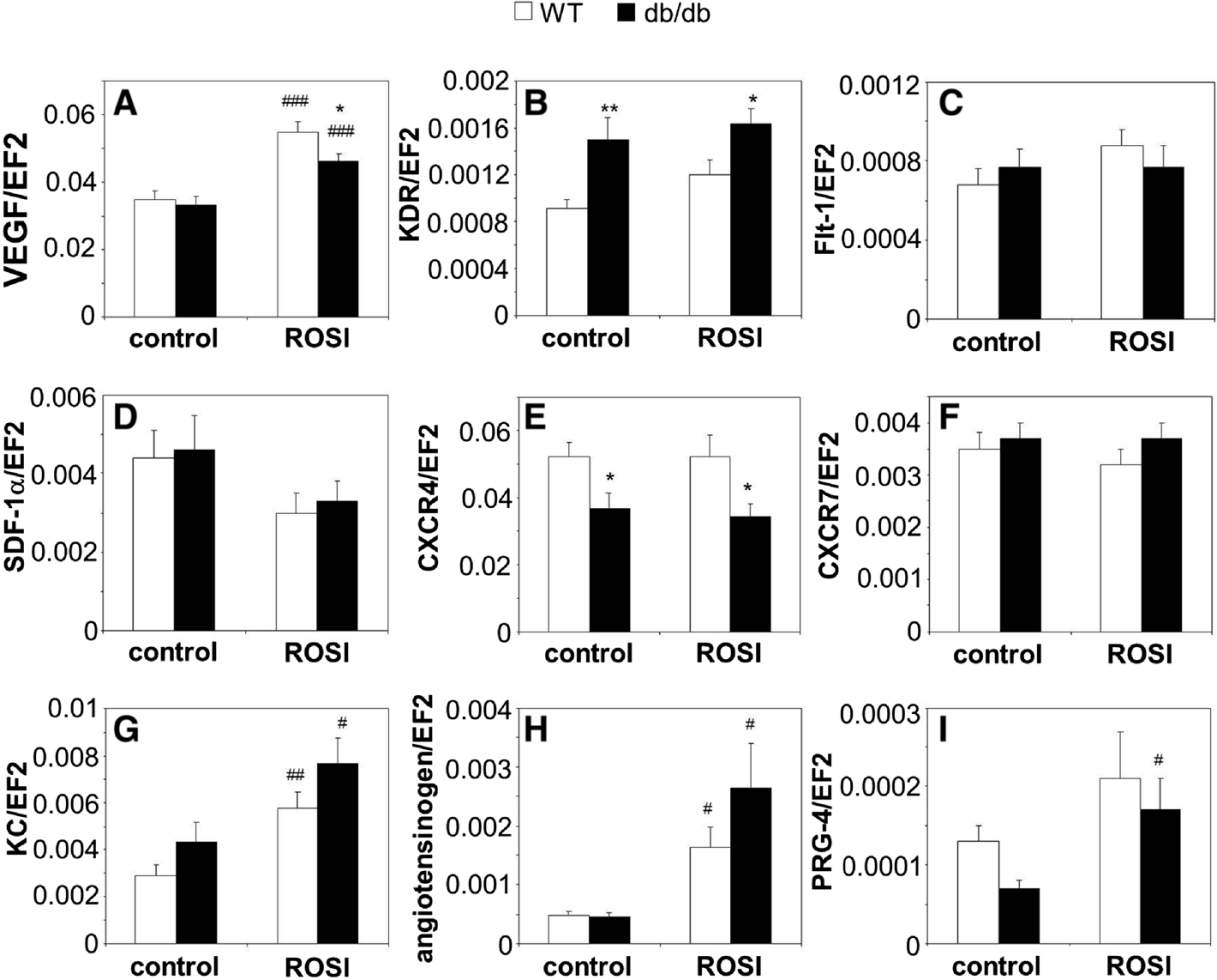
**Effect of PPARγ agonist rosiglitazone (ROSI, 10 μmol/L, 24 h) on expression of proangiogenic genes in PACs from wild type (WT) and diabetic (db/db) mice.** PACs cultured without ROSI are used as a control. **A**: VEGF. **B**: KDR. **C**: Flt-1. **D**: SDF-1. **E**: CXCR4. **F**: CXCR7. **G**: KC. **H**: Angiotensinogen. **I**: PRG-4. Quantitative RT-PCR. EF2 serves as an internal control. Each bar represents mean + SEM. N = 8-10, ^*^ *p* < 0.05, ^**^ *p* < 0.01 *versus* WT, ^#^ *p* < 0.05, ^##^ *p* < 0.01, ^###^ *p* < 0.001 *versus* control.

Next, we tested effects of rosiglitazone (10 μmol/L, 24 h) on expression of genes involved in angiogenesis, focusing on two major agents, VEGF and SDF-1. Expression of VEGF, similar in the cultured wild type and db/db PACs, was upregulated in response to rosiglitazone. This upregulation was slightly, but significantly weaker in cells isolated from diabetic mice (Figure 3A). However, we did not see changes on a protein level (data not shown). Expression of KDR (Flk-1, VEGFR-2), the major receptor mediating the proangiogenic VEGF action, was higher in db/db cells, and not modified by rosiglitazone (Figure 3B), while Flt-1 (VEGFR-1) level was affected neither by diabetes nor by rosiglitazone (Figure 3C). Like in the case of VEGF, expression of SDF-1 was similar in control wild type and db/db PACs. Treatment with rosiglitazone showed a tendency toward decrease in SDF-1 levels (Figure 3D), but this effect did not reach statistical significance (*p* = 0.06 in control PACs and *p* = 0.12 in db/db PACs). The same trend was observed for protein level (data not shown). Its major receptor, CXCR4 was, however, downregulated in db/db cells, the effect not modified by rosiglitazone (Figure 3E). Expression of CXCR-7 receptor was the same in all experimental groups (Figure 3F). Additionally, we found that both in wild type and db/db PACs rosiglitazone significantly increased production of KC, a potent proangiogenic chemokine (Figure 3G). Cells cultured with rosiglitazone showed also increased expression of two genes which may promote differentiation of proangiogenic precursors, namely angiotensinogen (Figure 3H) and proteolycan-4 (Figure 3I).

### Effect of therapy with rosiglitazone on PACs and endothelial cells in diabetic mice

Subsequently, we tested whether therapy with rosiglitazone would correct angiogenic functions of PACs and mature endothelial cells in diabetic mice. As expected, daily oral administration of rosiglitazone (10 mg/kg for 14 days) caused the increase in body mass by about 20% of initial values (Additional file 3: Figure S2A). Concomitantly, all measured biochemical parameters, like glucose, cholesterol, and triglycerides concentrations, together with percentage of glycated hemoglobin (HbA1c), were significantly elevated in diabetic animals and reduced after treatment with rosiglitazone (Additional file 3: Figure S2B-E). Hematological parameters of peripheral blood were not changed, apart from a total number of leukocytes, which was lower in db/db mice, regardless of rosiglitazone treatment (Additional file 1: Table S2).

**Figure 4:**
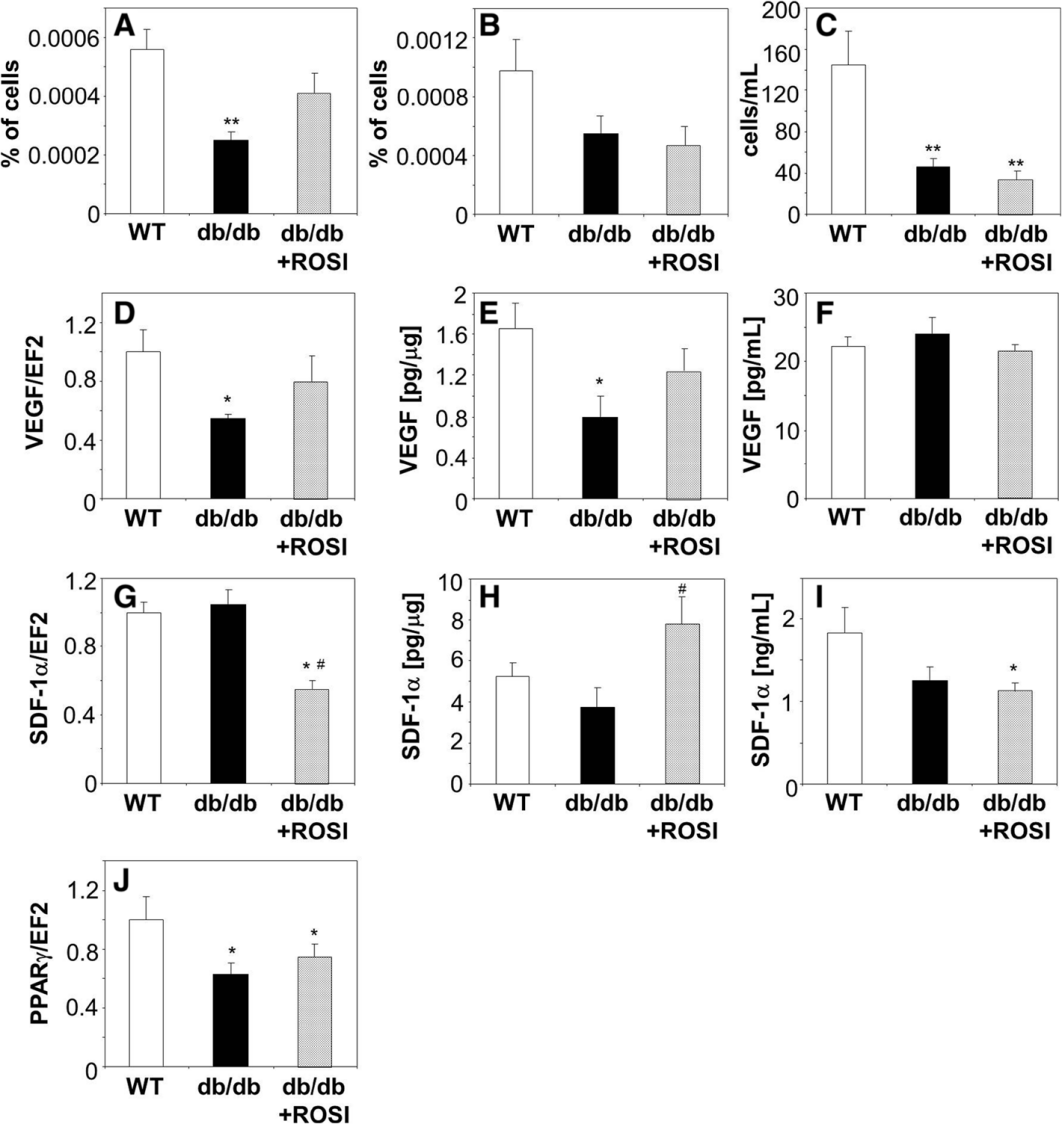
**Effect of oral daily administration of PPARγ agonist rosiglitazone (ROSI, 10 mg/kg of body weight, 14 days) on endothelial progenitor cells and expression of proangiogenic genes in diabetic (db/db) mice.** Control db/db and wild type (WT) mice were treated with vehicle. **A**: Percentage of CD45^−^KDR^+^Sca-1^+^ cells in bone marrow. **B**: Percentage of CD45^−^KDR^+^Sca-1^+^ cells in peripheral blood. **C**: Number of CD45^−^KDR^+^Sca-1^+^ cells in peripheral blood. Multicolor FACS phenotyping. **D**: Expression of VEGF mRNA in bone marrow. Quantitative RT-PCR. EF2 serves as an internal control. **E**: Concentration of VEGF protein in bone marrow. **F**: Concentration of VEGF protein in peripheral blood. ELISA. **G**: Expression of SDF-1 mRNA in bone marrow. Quantitative RT-PCR. EF2 serves as an internal control. **H**: Concentration of SDF-1 protein in bone marrow. **I**: Concentration of SDF-1 protein in peripheral blood. ELISA. **J**: Expression of PPARγ mRNA in bone marrow. Quantitative RT-PCR. EF2 serves as an internal control. Each bar represents mean + SEM. N = 8-10 (4A-C, F, I), N = 4-6 (4D, E, G, H, J), ^*^ *p* < 0.05, ^**^ *p* < 0.01, ^***^ *p* < 0.001 *versus* WT, ^#^ *p* < 0.05 *versus* db/db.

Flow cytometric analysis showed that endothelial progenitors (EPCs), defined as CD45^−^KDR^+^Sca-1^+^ cells, were significantly less frequent in the bone marrow of diabetic mice. Treatment with rosiglitazone resulted in a partial restoration of bone marrow EPCs pool (Figure 4A, *p* = 0.06), which was complete after prolongation of treatment time to 28 days (data not shown). Decreased frequency of CD45^−^KDR^+^Sca-1^+^ cells was found also in the peripheral blood of diabetic animals, although this tendency did not reach statistical significance (Figure 4B). The number of circulating EPCs was, however, strongly reduced in db/db mice and not affected by rosiglitazone (Figure 4A-C).

In parallel with changes in the bone marrow EPC pool, we observed a similar pattern (decrease in diabetic mice partially restored by rosiglitazone) in expression of VEGF, both at the mRNA and protein levels (Figure 4D,E). In contrast, concentration of VEGF in the blood was the same in all experimental groups (Figure 4F). On the other hand, expression of SDF-1 mRNA was the same in the bone marrow of wild type and db/db mice, but decreased in response to rosiglitazone (Figure 4G). Concentration of SDF-1 protein did not reflect, however, the level of mRNA expression and was increased in rosiglitazone treated mice (Figure 4H). This increase was not observed in the blood, where SDF-1 concentrations were lower in diabetic animals (Figure 4I). Noteworthy, expression of PPARγ in the bone marrow of diabetic db/db mice was reduced by approximately 50% and was not changed in response to rosiglitazone (Figure 4J).

We compared also the angiogenic potential of cultured PACs isolated from diabetic animals treated for two weeks with rosiglitazone with that from the untreated diabetic mice or wild type animals. Analysis of PAC migration and sprouting of capillaries from PAC spheroids showed the impaired activity of cells derived from diabetic animals, regardless of the treatment with rosiglitazone and despite the correction of metabolic status (data not shown). Similarly, paracrine potential of PACs and angiogenic activity of mature endothelial cells isolated from aorta were not rescued in diabetic mice treated with rosiglitazone (data not shown).

### Conditioned media from PACs improve blood flow restoration in diabetic mice

**Figure 5:**
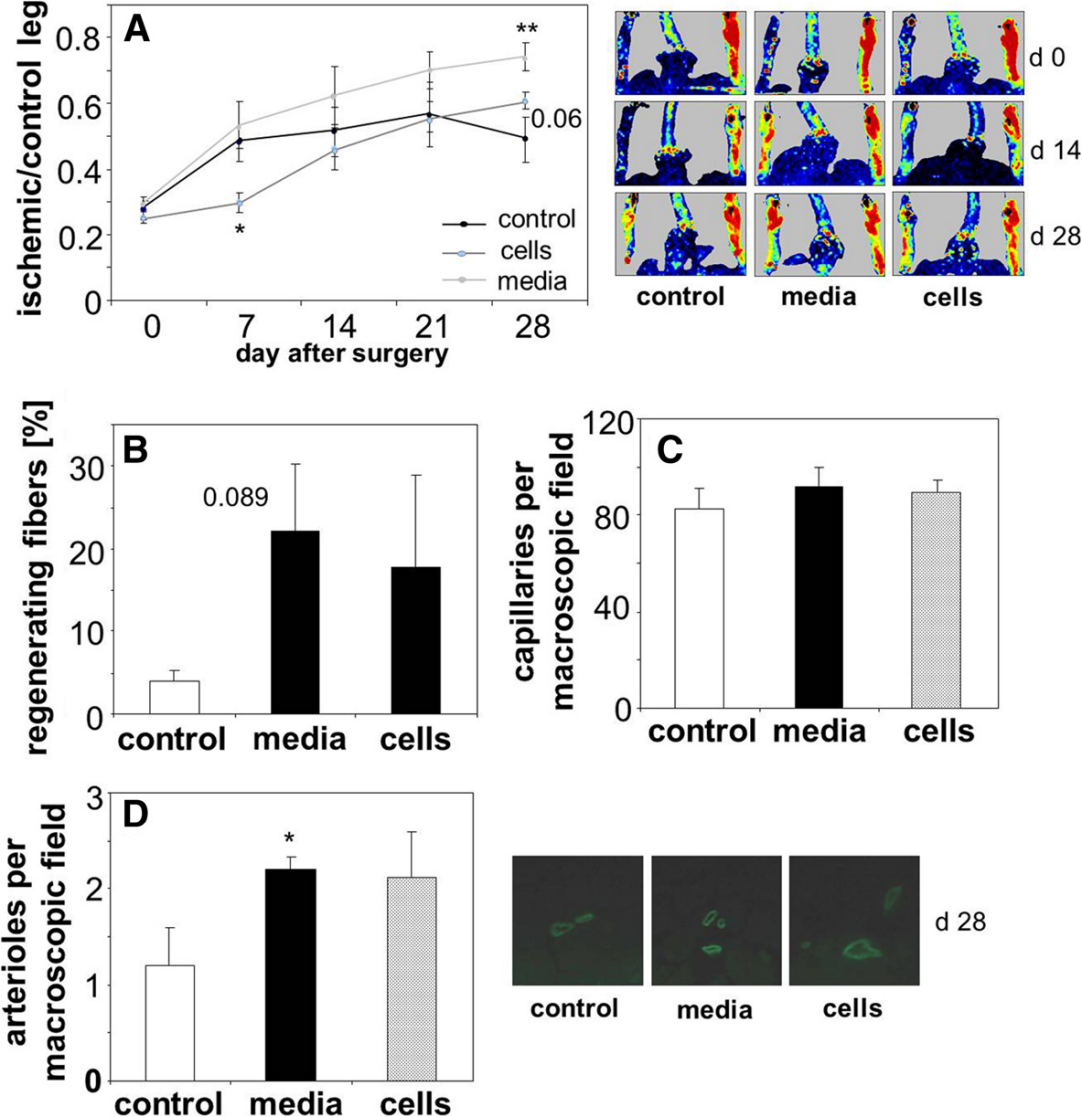
**Restoration of blood flow in ischemic muscles in diabetic (db/db) mice subjected to the hind limb ischemia.** Ischemic gastrocnemius was injected either with empty media (control, EBM-2 with 0.5% FBS), conditioned media (media, EBM-2 with 0.5% FBS harvested from cultured wild type PACs ), or PACs (cells, 2.5 x 10^5^ in control medium) 24 h after surgery (injections into 3 sites, total volume of 50 μL). **A**: Blood flow expressed as a ratio between ischemic and control leg. Numerical data and representative pictures. Laser-Doppler flowmetry. **B**: Percentage of regenerating fibers in gastrocnemius muscle, 28 days after surgery. Hematoxylin-eosin staining. **C**: Number of capillaries in gastrocnemius muscle, 28 days after surgery. Immunohistochemical staining for CD31. **D**: Number of arterioles in gastrocnemius muscle, 28 days after surgery. Immunohistochemical staining for αSMA. Numerical data and representative pictures. N = 4-6, ^*^ *p* < 0.05, ^**^ *p* < 0.01 *versus* control.

We checked whether PACs isolated from nondiabetic, wild type individuals may improve the blood flow restoration in ischemic muscles after hind limb ischemia in diabetic mice. To assess the importance of paracrine activity of PACs we compared effect of a) locally transplanted PACs and b) locally injected conditioned media harvested from PACs. Femoral artery ligation in db/db mice was followed either by injection of non-conditioned, empty medium ("control"), PACs isolated from syngeneic wild type mice and cultured *in vitro* ("cells"), or conditioned media harvested from such cells ("media"). Measurements of perfusion done immediately after surgery (day 0) and on days 7^th^, 14^th^, 21^st^, and 28^th^ showed that blood flow recovery in control diabetic mice was not effective, as on day 28^th^ it still did not reach a 50% of perfusion measured before surgery. However, this process was enhanced in animals injected with conditioned media on day 28^th^, what confirms the importance of paracrine influence of bone marrow derived cells. Transplantation of PACs was less effective, or could even slow down the recovery from ischemia at early time points (Figure 5A). There was also a tendency toward a higher proportion of regenerating fibers in the group injected with conditioned media in comparison to control group (Figure 5B), not significant in the group subjected to PAC transplantation (*p* > 0.3). Immunohistochemical analyzes performed on day 28^th^ showed no differences in density of CD31-positive capillaries between experimental groups (Figure 5C). However, there was a higher number of αSMA-positive arterioles in animals treated with conditioned media and a similar tendency in the group after PAC transplantation (Figure 5D). No statistically significant changes in frequency or number of CD45^−^KDR^+^Sca-1^+^ cells were found at this time point either in bone marrow or in peripheral blood (data not shown).

### Gene expression profiles of PACs

To better understand the effect of diabetes on proangiogenic potential of the bone marrow-derived cells we compared a transcriptome of PACs isolated from the wild type and db/db mice. Additionally, for cells of both genotypes cultured *in vitro*, we checked the effect of hypoxia, the most important proangiogenic stimulus. Results are summarized in Table 1. Generally, the influence of hypoxia (2% O_2_ for the final 24 h incubation period) on gene expression was similar in the wild type and db/db cells, mainly leading to upregulation of genes involved in energetic metabolism, especially in glycolysis.

**Table 1:** **Analysis of transcriptome of PACs isolated from bone marrow of wild type** (**WT**) **or diabetic** (**db**/**db**) **mice and cultured** ***in vitro*** **for 9 days**

**Gene**	**Normoxia**		**Hypoxia**	
**Oxidative stress and cytoprotection**	**WT**	**db** **/** **db**	**WT**	**db** **/** **db**
**Nox4** (NOX4)	148 ± 20.3	245 ± 15.2*	153 ± 25.0	302 ± 123.8^#^0.082
**Cyba** (p22phox)	29331 ± 1383.1	35829 ± 1277.4**	28248 ± 2195.8	34380 ± 5610.4
**Prkcd** (PKCδ)	3134 ± 75.8	3698 ± 286.6*	3667 ± 518.5	3965 ± 413.3
**Chst1** (GST-1)	6979 ± 411.2	4412 ± 341.3**	8339 ± 562.9	5774 ± 374.8*^#^
**Mgst3** (GST III)	3388 ± 453.1	1741 ± 100.7*	4076 ± 590.9	2563 ± 342.6* 0.070; ^#^0.091
**Gpx4** (GPX4)	7819 ± 354	6920 ± 423.3*	8394 ± 76.9^#^	7599 ± 614.9* 0.076; ^#^0.099
**Inflammation**	**WT**	**db** **/** **db**	**WT**	**db**/**db**
**Tnfrsf1a** (TNFR-1)	1259 ± 68.7	1447 ± 124.7* 0.051	1103 ± 298.5	1147 ± 82.5^#^
**Csf3r** (G-CSF3R)	188 ± 6.7	214.9 ± 9.5*	209 ± 7.9	237 ± 36.1
**Cxcl16** (CXCL16)	2328 ± 215.3	3194 ± 403.5*	2464 ± 319.2	2972 ± 535.2
**Selplg** (P-selectin ligand)	1930 ± 219.1	2758 ± 324.4*	2737 ± 822.8	3214 ± 311.0^#^ 0.077
**Extracellular matrix remodeling**	**WT**	**db** **/** **db**	**WT**	**db** **/** **db**
**Mmp2** (MMP-2)	2825 ± 395.2	6498 ± 1373.3*	2567 ± 900.6	6454 ± 905.8**
**Mmp9** (MMP-9)	1906 ± 1005.0	7554 ± 4171.7* 0.069	1713 ± 637.0	9625 ± 955.3***
**Mmp14** (MMP-14)	5081 ± 513.8	5965 ± 461.9*	5499 ± 518.5	5758 ± 519.5
**Mmp23** (MMP-23)	1625 ± 273.8	2127 ± 267.9*	2396 ± 521.2^#^0.054	2956 ± 338.2^#^
**Migration and proliferation**	**WT**	**db** **/** **db**	**WT**	**db** **/** **db**
**Itgam** (integrin-α1)	983 ± 254.4	1397 ± 30.8* 0.052	1499 ± 313.8^#^0.096	1397 ± 498.6*
**Itgb2** (integrin-β2)	354 ± 37.4	438 ± 30.9*	472 ± 76.2^#^	587 ± 90.8* 0.085^#^
**Itgb4** (integrin-β4)	105 ± 11.2	141 ± 4.28*	178 ± 66.0^#^ 0.096	187 ± 26.7^#^
**Ngef** (NGEF)	163 ± 2.9	104 ± 8.2**	246 ± 36.2^#^	140 ± 5.7*^##^
**Cct2** (CCT2)	166 ± 4.0	139 ± 4.9**	136 ± 11.6^#^	145 ± 16.6
**Msx1** (MSX-1)	184 ± 18.5	132 ± 13.7**	159 ± 17.8^#^0.081	127 ± 1.24*
**Cdkl2** (CDKL2)	233 ± 17.3	177 ± 7.4**	205 ± 19.7^#^0.066	193 ± 15.9
**Areg** (AREG)	322 ± 69.7	118 ± 14.5*	290 ± 126.6	155 ± 39.4* 0.099
**Mdk** (midkin)	236 ± 6.9	313 ± 21.6**	184 ± 3.1^###^	300 ± 17.9**
**Nos3** (NOS3)	122 ± 3.0	142 ± 5.2**	99 ± 7.4^#^	106 ± 6.3^###^
**Cav1** (caveolin-1)	4279 ± 675.2	2171 ± 242.9*	6460 ± 418.0^##^	4889 ± 968.4*^#^
**Fgf7** (FGF-7)	7073 ± 780.0	5858 ± 795.5* 0.066	5736 ± 403.8^#^	5306 ± 958.3
**Ang2** (angiogenin)	198 ± 50.5	138 ± 15.9* 0.085	164 ± 42.4	131 ± 6.1
**Vegfb** (VEGF-B)	1637 ± 107.1	1641 ± 91.4	2396 ± 97.9^###^	2195 ± 246.8^#^
**Vegfc** (VEGF-C)	714 ± 69.2	524 ± 91.5*	730 + 111.0	904 ± 331.3^#^ 0.089
**Vegfd** (VEGF-D)	791 ± 50.7	499 ± 105.6*	1011 ± 289.9	839 ± 113.8^##^

For last 24 h cell were incubated in normoxia (21% O2) or hypoxia (2% O2).

Means ± SEM of normalized fluorescence signal. N = 3 for each gene, *p < 0.05, **p < 0.01 WT *versus* db/db, ^#^ *p* < 0.05, ^##^ *p* < 0.01, ^###^ *p* < 0.001 normoxia *versus* hypoxia. For 0.05 < *p* < 0.1 exact *p* values are shown.

Comparison of wild type and db/db PACs cultured in normoxia revealed that ~700 transcripts were differentially expressed (*p* < 0.05), including 54 transcripts with more than two-fold difference (*p* < 0.01). Analysis of molecular pathways using GeneGO platform indicated that db/db PACs displayed higher expression levels of pro-oxidative genes, such as NADPH-4 oxidase (Nox4), p22phox subunit, or protein kinase C-δ (PKCδ). It was accompanied by lower levels of cytoprotective genes, such as glutathione S-transferases (GST-1 and GST-3) or glutathione peroxidase-4 (GPX-4) (Table 1). PACs isolated from diabetic mice had also increased expression of proinflammatory genes, e.g. tumor necrosis factor receptor-1 (TNFR-1), granulocyte-colony stimulating factor-3 receptor (G-CSF-3R), CXCL16 chemokine, or P-selectin ligand (Table 1). Similarly, genes associated with remodeling of extracellular matrix, such as MMP-2, MMP-9, MMP-14 and MMP-23 matrix metalloproteinases were upregulated in db/db cells (Table 1). Diabetic PACs had also higher expression of integrin-α1, −β2 and -β4 mRNAs, with concomitant lower expression of genes responsible for filopodia formation, like efexin (NGEF) or T-complex protein-1 subunit-β (CCT2) (Table 1), what might be associated with the impaired migration capacity of db/db cells. Importantly, expressions of proangiogenic genes, such as fibroblast growth factor-7 (FGF-7), VEGF-C, VEGF-D, or angiogenin, were decreased in PACs isolated from diabetic animals (Table 1).

### Expression of PPARγ in PACs

**Figure 6:**
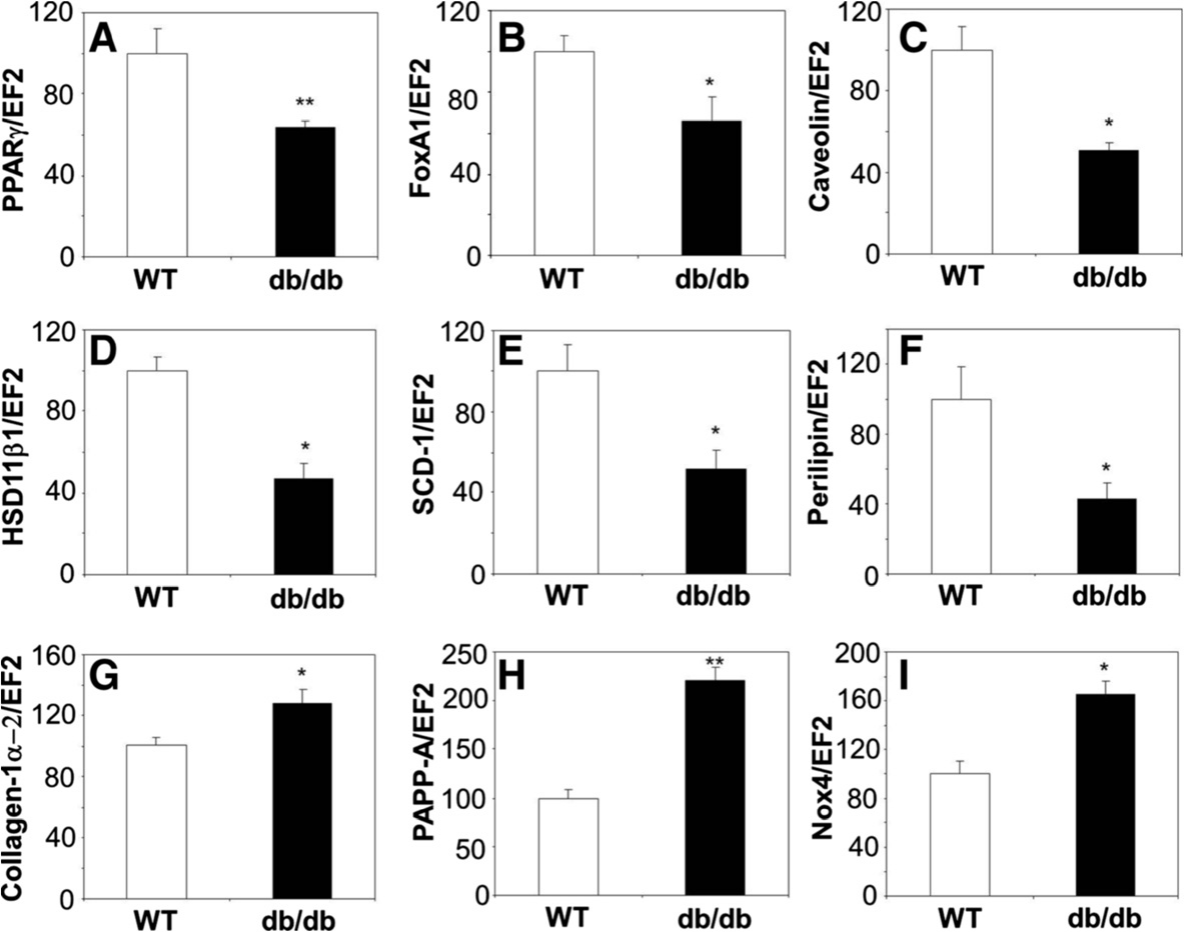
**Expression of PPARγ associated genes in PACs from diabetic (db/db) mice, shown as a percentage of gene expression in PACs from wild type (WT) mice. A**: PPARγ. **B**: FoxA1. **C**: caveolin-1. **D**: HSD11β. **E**: SCD-1. **F**: perilipin. **G**: collagen-1α. **H**: PAPP-A. **I**: Nox4. Quantitative RT-PCR. EF2 serves as an internal control. Each bar represents mean + SEM. N = 3, ^*^ *p* < 0.05, ^**^ *p* < 0.01 *versus* WT.

Transcriptome analysis showed that PACs isolated from db/db mice had reduced expression of PPARγ (Figure 6A). This decrease seems to have a functional effect, as suggested by changes in expression of PPARγ targeted genes (Figure 6). We found 8 genes known to be directly regulated by PPARγ [37], which were differently expressed in PACs from wild type and db/db mice. Interestingly, expressions of genes positively regulated by PPARγ [38-42], namely forkhead box-A1 (FoxA) (Figure 6B), caveolin-1 (Figure 6C), 11β-hydroxysteroid dehydrogenase type 1 (HSD11β1) (Figure 6D), stearoyl-CoA desaturase-1 (SCD-1) (Figure 6E), and perilipin (Figure 6F) were decreased in db/db PACs. In contrast, expression of genes negatively regulated by PPARγ [43,44], namely collagen-1α2 (Figure 6G), pregnancy associated plasma protein-A (PAPP-A) (Figure 6H), and Nox4 (Figure 6I) were increased in db/db PACs.

### PPARγ heterozygosity in normoglycemic mice does not impair PAC functions

Proper level of PPARγ is necessary for development of placenta vasculature [30,45], pointing to role of PPARγ in angiogenesis. In diabetic db/db mice impaired angiogenesis and decrease in number of PACs in bone marrow or peripheral blood is accompanied by the reduced expression of PPARγ both in PACs (Figure 6A) and in bone marrow (Figure 4J). Therefore one can hypothesize that downregulation of PPARγ expression may be a primary cause of the impairment in PAC or endothelial cell angiogenic potential.

**Figure 7:**
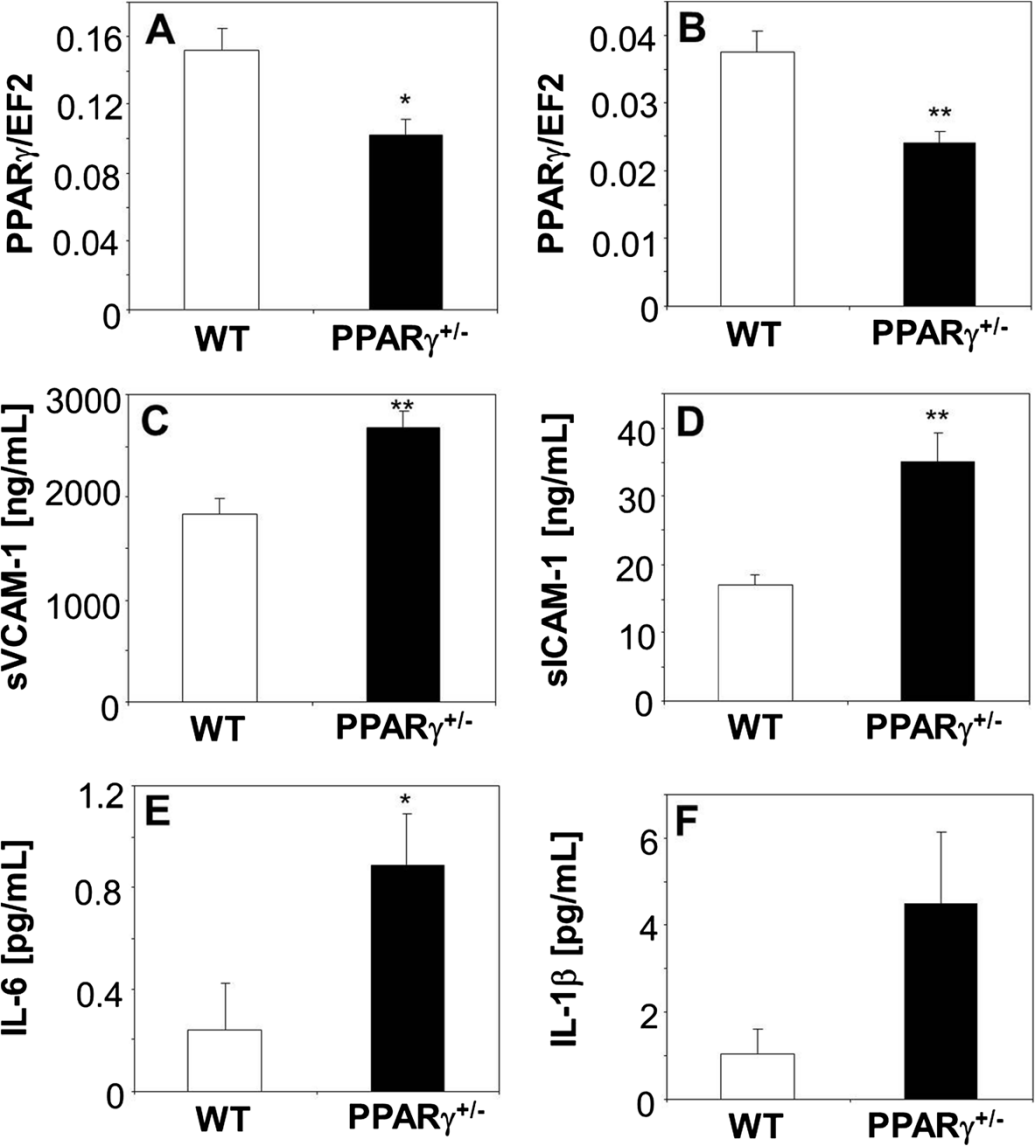
**Proinflammatory phenotype of PPARγ haplodeficient mice. A**-**B**: Expression of PPARγ in wild type (WT) and PPARγ heterozygotic (PPARγ^+/−^) mice. **A**: bone marrow. **B**: PACs. Quantitative RT-PCR. EF2 serves as an internal control. **C-F**: Concentration of inflammatory mediators in the blood plasma of WT and PPARγ-HT mice. **C**: sVCAM-1. **D**: sICAM-1. **E**: IL-6. **F**: IL-1β. Milliplex®MAP platform. Each bar represents mean + SEM. N = 4-6, ^*^ *p* < 0.05, ^**^ *p* < 0.01 *versus* WT.

To verify this supposition, in the last set of experiments we investigated angiogenesis in normoglycemic mice carrying one dysfunctional allele of PPARγ (PPARγ^+/−^). Such animals had a decreased PPARγ mRNA expression both in the bone marrow and cultured PACs (Figure 7A,B), to a similar extent as in db/db mice. They also had a more proinflammatory phenotype, as illustrated by higher concentrations of sVCAM-1, soluble intercellular adhesion mulecule-1 (sICAM-1), IL-6, and IL-1β in the blood (Figure 7C-F). No differences were found, however, between expressions of genes regulating angiogenic activity (VEGF, KDR, SDF-1, and CXCR4) in PACs isolated form PPARγ^+/+^ and PPARγ^+/−^ animals (Additional file 4: Figure S3A-D). Also frequency of CD45^−^KDR^+^Sca-1^+^ endothelial precursors were similar in both groups (Additional file 4: Figure S3E-F). Based on transcriptome analysis of PACs isolated from WT and db/db mice we selected a candidate genes to verify effects of PPARγ haplodeficiency and hypoxia on their expression. Cells isolated from PPARγ^+/−^ animals did not display different expression levels of pro-oxidative genes (Nox4, p22phox), nor lower levels of cytoprotective genes (GST-3, Table 2). Despite lower expression of PPARγ in such cells, than in WT ones they were characterized by the same mRNA amount of proinflammatory genes (TNFR-1, P-selectin ligand) and matrix metalloproteinases MMP-2, MMP-9 (Table 2). Similarly, genes associated with proangiogenic properties of PACs (VEGF, SDF-1 and their receptors) were expressed at the same level in PPARγ^+/+^ and PPARγ^+/−^ cells. However, CAV1 was upregulated in PPARγ^+/−^ PAC (Table 2).

**Table 2:** **Analysis of gene expression in PACs isolated from bone marrow of wild type** (**PPARγ** ^+/+^) **or PPARγ haplodeficient mice** (**PPARγ** ^+/−^) **and cultured** ***in vitro*** **for 9 days**

**Gene**	**Normoxia**		**Hypoxia**	
**Oxidative stress and cytoprotection**	**PPARγ** ^**+/+**^	**PPARγ** ^**+/−**^	**PPARγ** ^**+/+**^	**PPARγ** ^**+/−**^
**Nox4** (NOX4)	0,00065 ± 0,00012	0,00090 ± 0,00016	0,00052 ± 0,00008	0,00129 ± 0,00023*
**Cyba** (p22phox)	0,702 ± 0,102	0,748 ± 0,094	0,449 ± 0,077	0,829 ± 0,185
**Mgst3** (GST III)	0,043 ± 0,005	0,060 ± 0,006*	0,043 ± 0,004	0,060 ± 0,005*
**Inflammation**	**PPARγ** ^**+/+**^	**PPARγ** ^**+/−**^	**PPARγ** ^**+/+**^	**PPARγ** ^**+/−**^
**Tnfrsf1a** (TNFR-1)	0,178 ± 0,028	0,235 ± 0,017* 0.092	0,132 ± 0,015	0,173 ± 0,012* 0.051^##^
**Selplg** (P-selectin ligand)	0,00018 ± 0,00008	0,00021 ± 0,00005	0,00019 ± 0,00004	0,00031 ± 0,00006
**Extracellular matrix remodeling**	**PPARγ** ^**+/+**^	**PPARγ** ^**+/−**^	**PPARγ** ^**+/+**^	**PPARγ** ^**+/−**^
**Mmp2** (MMP-2)	0,070 ± 0,005	0,086 ± 0,010	0,044 ± 0,003^##^	0,101 ± 0,029* 0.081
**Mmp9** (MMP-9)	0,416 ± 0,124	0,194 ± 0,067	0,263 ± 0,067	0,148 ± 0,033
**Migration and proliferation**	**PPARγ** ^**+/+**^	**PPARγ** ^**+/−**^	**PPARγ** ^**+/+**^	**PPARγ** ^**+/−**^
**Nos3** (NOS3)	0,00049 ± 0,00006	0,00063 ± 0,00013	0,00044 ± 0,00005	0,00061 ± 0,00009
**Cav1** (caveolin-1)	0,051 ± 0,006	0,080 ± 0,007**	0,104 ± 0,010^##^	0,162 ± 0,008**,^###^
**Sdf**-**1** (CXCL12)	0,069 ± 0,021	0,110 ± 0,022	0,071 ± 0,022	0,088 ± 0,010
**Cxcr4** (CXCR-4)	0,023 ± 0,007	0,023 ± 0,004	0,033 ± 0,006	0,037 ± 0,004#
**Cxcr7** (CXCR-7)	0,0021 ± 0,0005	0,0021 ± 0,0003	0,0095 ± 0,0020^#^	0,0081 ± 0,0013^##^
**Vegfa** (VEGF-A)	0,052 ± 0,005	0,051 ± 0,005	0,185 ± 0,047^#^	0,164 ± 0,008^###^
**Flt**-**1** (VEGFR-1)	0,00028 ± 0,00004	0,00038 ± 0,00011	0,00045 ± 0,00003^#^	0,00065 ± 0,00019
**KDR** (VEGFR-2)	0,0021 ± 0,0002	0,0027 ± 0,0007	0,0013 ± 0,0005	0,0019 ± 0,0003

For last 24 h cell were incubated in normoxia (21% O_2_) or hypoxia (2% O_2_). Quantitative RT-PCR, EF2 serves as an internal control. Means ± SEM. N = 5-6, *p < 0.05, **p < 0.01 PPARγ^**+/+**^ *versus* PPARγ^**+/−**^, ^#^ *p* < 0.05, ^##^ *p* < 0.01, ^###^ *p* < 0.001 normoxia *versus* hypoxia. For 0.05 < *p* < 0.1 exact *p* values are shown.

**Figure 8:**
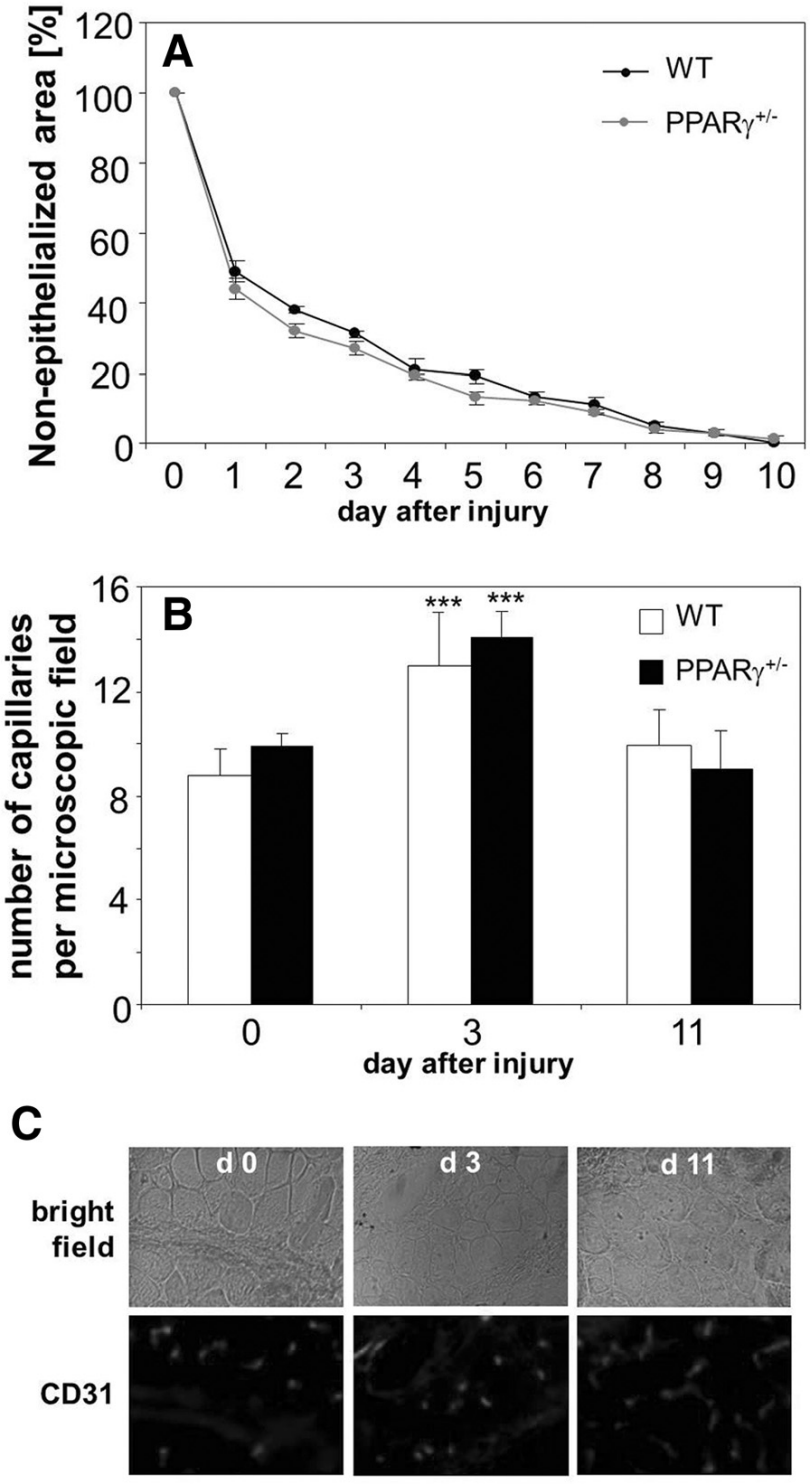
**Healing of cutaneous wounds in wild type (WT) and PPARγ haplodeficient (PPARγ** ^**+/−**^ **) mice. A**: Closure of wounds expressed as percentage of non-epithelialized area. Surface of wound measured immediately after surgery was taken as 100%. **B**: Number of capillaries stained for CD31 antigen in the muscles located under healthy skin (day 0) and under wounded skin, 3 and 11 days after surgery. **C**: Representative pictures showing CD31 immunostaining. Fluorescence microscopy. Mean + SEM. N = 4-5, ***p < 0.001 *versus* day 0.

In earlier experiments we have demonstrated that angiogenesis induced in response to skin injury is impaired in the db/db diabetic mice, what is accompanied by defective wound healing [31]. Here we performed similar experiment using PPARγ^+/+^ and PPARγ^+/−^ animals subjected to full thickness skin excision. Reduced expression of PPARγ influenced neither skin wound closure rate (Figure 8A) nor injury-induced increase in number of capillaries within the wounded tissue (Figure 8B,C).

Then, we compared the efficacy of angiogenesis employing the model of blood flow restoration in PPARγ^+/+^ and PPARγ^+/−^ animals subjected to hind limb ischemia (Additional file 5: Figure S4A-H). Femoral artery ligation did not affect expression of VEGF in bone marrow or ischemic muscle (Additional file 5: Figure S4A,B), as demonstrated by qRT-PCR analysis performed on day 1 after surgery. In the same time, the expression of SDF-1 did not change significantly in the bone marrow (Additional file 5: Figure S4C), but was upregulated in the muscle (Additional file 5: Figure S4D). The muscular levels of KDR were similar before and one day after artery ligation (Additional file 5: Figure S4E), whereas expression of CXCR4 showed a tendency toward upregulation (Additional file 5: Figure S4F, *p* = 0.08 and *p* = 0.09 for PPARγ^+/+^ and PPARγ^+/−^ mice, respectively).

**Figure 9:**
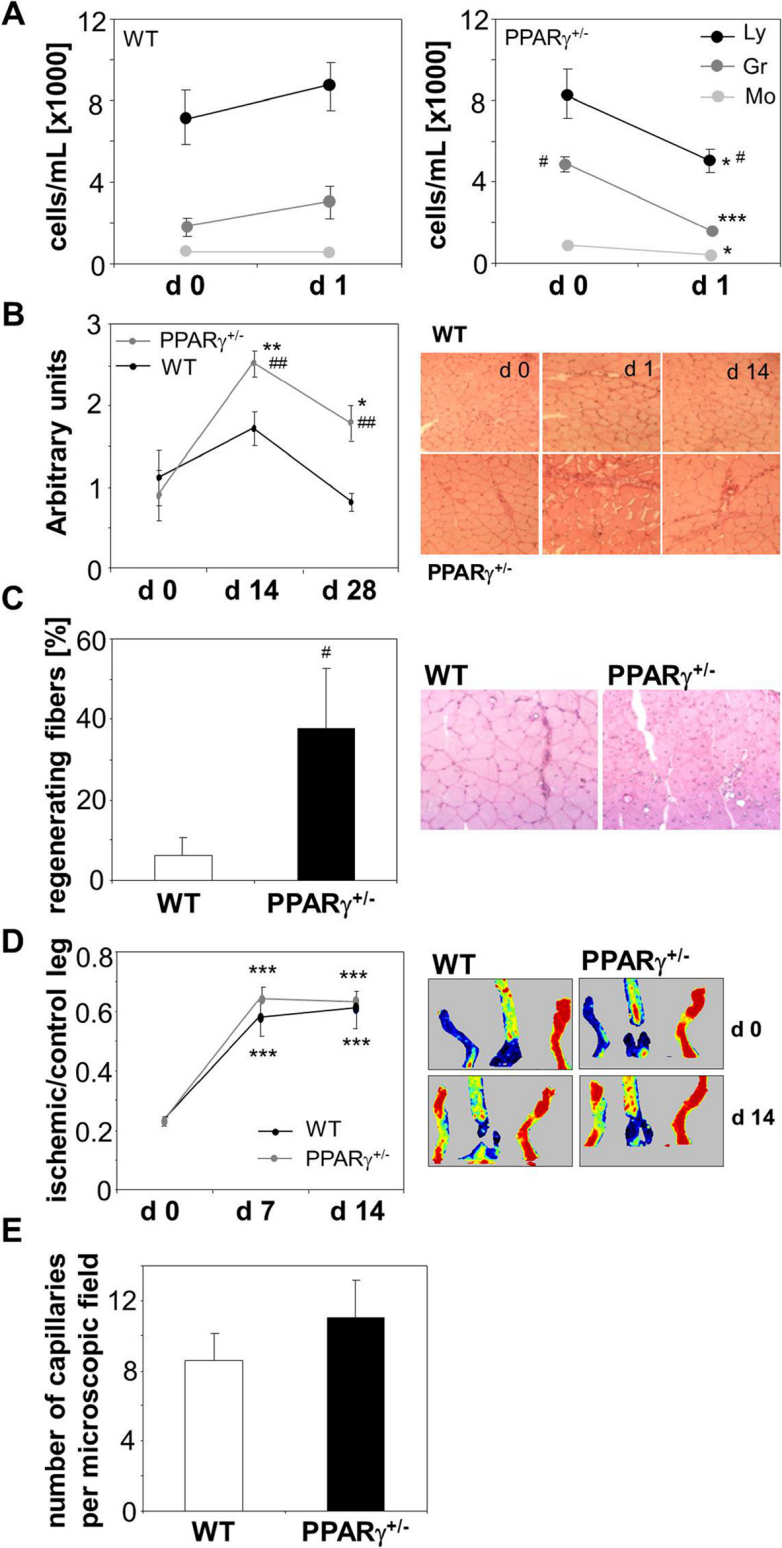
**Effect of induction of hind limb ischemia in wild type (WT) and PPARγ haplodeficient (PPARγ** ^**+/−**^ **) mice. A**: Number of lymphocytes (Ly), granulocytes (Gr) and monocytes (Mo) in the peripheral blood of WT and PPARγ^+/−^ mice before (d 0) and 1 day (d 1) after surgery. Automated hematology analyzer. **B**: Leukocytic infiltration of gastrocnemius muscle before (d 0) and 14 and 28 days (d 14 and d 28) after surgery. Semiquantitative analysis and representative pictures. Hematoxylin-eosin staining. **C**: Percentage of regenerating fibers in ischemic muscle, 28 days after surgery. Numerical data and representative picture. Hematoxylin-eosin staining. **D**: Blood flow expressed as a ratio between ischemic and control leg. Numerical data and representative pictures. Laser-Doppler flowmetry. **E**: Number of capillaries in the ishcmic muscle, 28 days after surgery. Immunohistochemical staining for CD31. Mean + SEM. N = 5-9 (9A-C, E), N = 10 (9D), *p < 0.05, **p < 0.01, ***p < 0.001 *versus* d 0; ^#^ *p* < 0.05, ^##^ *p* < 0.01, ^###^ *p* < 0.001 *versus* WT.

We found some differences between genotypes in response to hind limb ischemia. First, untreated PPARγ^+/−^ individuals had a higher number of circulating granulocytes, whereas numbers of monocytes and lymphocytes were similar in both experimental groups. One day after femoral artery ligation numbers of monocytes and granulocytes in the peripheral blood decreased significantly in PPARγ^+/−^ mice, but remained stable in their wild type counterparts (Figure 9A). This was accompanied by significantly higher leukocytic infiltration of ischemic muscle in the PPARγ^+/−^ individuals, observed on day 1^st^ and 14^th^ after artery ligation (Figure 9B). Second, we found more regenerating muscle fibers, with centrally located nuclei, on day 14^th^ after induction of ischemia in mice with lower expression of PPARγ (Figure 8C). Nevertheless the restoration of blood flow in ischemic muscle monitored on day 1^st^, 7^th^, and 14^th^, as well as number of capillaries counted on day 14^th^ were the same, regardless of PPARγ genotype (Figure 9D,E).

## Discussion

Angiogenic potential of PACs and mature endothelial cells is impaired in db/db mice. We demonstrated that it can be restored by activation of PPARγ in cells cultured with rosiglitazone. We also found that diabetes is associated with reduced expression of PPARγ. Thus, one could hypothesize that decreased level of PPARγ may contribute to the inhibition of neovascularization observed in diabetic individuals. Experiments performed in normoglycemic PPARγ haplodeficient mice showed, however, that this is not the case and reduced expression of PPARγ does not affect angiogenesis.

Dysfunction and disturbed angiogenic potential of endothelial cells in diabetes have been comprehensively studied [4]. Much less is known about effect of diabetes on vasculogenic activities of the bone marrow-derived cells. We compared angiogenic potential of PACs, the mononuclear cells isolated from bone marrow of wild type or db/db mice and cultured under conditions promoting endothelial differentiation. Our analysis showed that cells derived from db/db mice displayed reduced angiogenic capacities. Especially, they demonstrated impaired migration, formation of cord-like structures on Matrigel and capillary outgrowth from spheroids. Only the proliferation rate was not significantly affected. Our results are in accordance with other studies showing decreased angiogenic potential of diabetic EPC isolated both from humans and animals [13-17]. Recently published paper by Li et al., demonstrated that advanced glycation end products (AGE) that are present in diabetes induce EPC apoptosis, impair SDF-1 and NO production [46]. Deleterious effects of diabetes was also reported for mesenchymal stem cells in humans [47].

Transcriptome analysis suggests a reduced capacity of db/db PACs to counteract ROS production. The results may also suggest that their impaired migration can be associated with upregulation of integrins accompanied by downregulation of genes involved in filopodia formation, such as efexin or CCT2 [48]. Furthermore, PACs isolated from db/db mice showed decreased paracrine potential, what might be related to decreased expression of VEGF-C, VEGF-D, FGF7 or angiogenin.

It has been demonstrated that PPARγ ligands influence angiogenesis, acting generally as proangiogenic agents, and increasing number of bone marrow derived endothelial progenitors. In animal models, such as diabetic rats, treatment with pioglitazone increased density of capillaries in the heart and skeletal muscle by a mechanism unrelated to VEGF-mediated angiogenesis [5]. Proangiogenic effect of pioglitazone, associated with accelerated healing of ulcers, was also observed in normoglycemic rats [49]. What is more, pioglitazone prevented EPC from apoptosis *via* PI3K/Akt signaling pathway [50]. Yet another study proved that beneficial effects of PPARγ activation might be also obtained by using other compounds. A dual PPARα/γ agonist, aleglitazar, administrated to mice at a 10 mg/kg/day dose increased the number and function of circulating angiogenic cells. Furthermore, aleglitazar reduced oxidative stress-induced apoptosis and p53 expression, while phosporylation of eNOS and Akt was elevated [51]. On the other hand, a deleterious effect on EPC biology was demonstrated for too strong PPARγ activation by palmitic acid. Palmitic acid is a natural PPARγ ligand which concentration increases in diabetes leading to impairment of EPC migration and proliferation by PPARγ-mediated STAT5 transcription inhibition [52]. Importantly, improved function of EPC and endothelial cells were described in diabetic patients under rosiglitazone therapy [19,20], the effect resulting possibly from reduced generation of ROS and improved bioavailability of NO [19]. In individuals with diabetes it is not easy, however, to discriminate the effects of metabolic correction and direct regulation of angiogenic capacities of endothelial or bone marrow-derived cells.

In our studies, to activate PPARγ *in vitro* in PACs we used rosiglitazone at the dose of 10 μmol/L. As demonstrated by Cox et al., a single oral dose of rosiglitazone (8 mg) in humans leads to a maximal concentration of 603 ng/mL (1.69 μmol/L) in the plasma [53]. Rosiglitazone is one of three thiazolidinediones introduced in the treatment of type 2 diabetes. Rosiglitazone provided a sustained glycemic control and seemed able to slow down the progression towards T2DM in patients with impaired glucose tolerance [54]. We demonstrated a direct effect of PPARγ activation on PACs angiogenic potential. Incubation of db/db PACs with rosiglitazone *in vitro*, improved their migration, formation of cord-like structures and capillary outgrowth. These effects were probably PPARγ-dependent, as were prevented by preincubation of cells with GW9662, PPARγ antagonist. Similar increase in migratory capacities of cultured bone marrow derived cells was observed earlier after treatment with pioglitazone or troglitazone [21,22,26] which may be mediated by reduced expression of ICAM-1 and VCAM-1 adhesion molecules [55]. Beneficial effects of PPARγ activation by rosiglitazone was also proved for vascular smooth muscle cells. Rosiglitazone treatment resulted in increased protein kinase G activity leading to attenuated hyperplasia after vascular injury [56].

There are, however, studies showing the antiangiogenic activity of PPARγ. In fact, in the first report, Murata et al. described a significant decrease in migration and proliferation of choroidal endothelial cells exposed to troglitazone or rosiglitazone [57]. Similarly, troglitazone suppressed HUVEC migration, the effect not reversed by GW9662, so possibly independent of PPARγ [27]. Also KR-62980, another PPARγ agonist reduced HUVEC migration and formation of cord-like structures on Matrigel, which was accompanied by apoptotic cell death [28]. The reason of such discrepancies are not clear. One can suppose that concentration of PPARγ ligands used in some experiments was high and therefore proapoptotic [27,28,57]. Another possibility is that ligands of PPARγ might have different effect on endothelial cells from different vascular beds. The well characterized PPARγ target gene is CD36, receptor for antiangiogenic thrombospondin-1 (TSP1), and its upregulation by PPARγ agonist may inhibit endothelial functions [58]. CD36 is expressed mainly on microvascular endothelial cells, and at lower level on venous endothelium [59]. Moreover, even in microvasculature the expression of CD36 is organ specific, with the highest level in the heart, muscle and lungs and very low in the bone marrow [60]. One could suppose that such differences may modify the angiogenic response to PPARγ agonists.

We also showed that *in vivo* administration of rosiglitazone partially restored EPC population in the bone marrow, although not in the peripheral blood. Analysis of gene expression revealed that db/db mice have a decreased level of VEGF and unchanged SDF-1 in the bone marrow. In such animals, rosiglitazone showed only a tendency toward upregulation of VEGF, but significantly affected SDF-1. Concentration of SDF-1 protein in the bone marrow increased, while remained unchanged in peripheral blood, thus the gradient of SDF-1 possibly was enhanced. The results were, however, complex: increase in concentration of SDF-1 protein in the bone marrow was not reflected by expression of SDF-1 mRNA, which was even lower after rosiglitazone administration. This suggests that rosiglitazone may influence SDF-1 not primarily at the mRNA level. Similar discrepancy between SDF-1 mRNA and protein expression was observed, for instance, in murine bone marrow after stimulation with granulocyte colony stimulating factor (G-CSF) [61]. Here, the possible mechanism was ascribed to regulation of proteases which are known to degrade SDF-1, such as MMP-9, MMP-2, neutrophil elastase or cathepsin G [60]. Indeed, MMP-9 and MMP-2 are inhibited by PPARγ agonists [27]. Similarly to our finding a decreased expression of SDF-1 was detected in magnetically sorted EPC cells isolated from bone marrow of diabetic mice [62].

Bone marrow derived cells may facilitate angiogenesis, mainly through paracrine effects. Our *in vitro* experiments revealed that paracrine activity of PACs isolated from db/db mice is impaired. Therefore we checked whether the wild type cells may be useful in therapeutic neovascularization and restoration of proper blood perfusion in ischemic muscles of diabetic animals. To this aim we induced hind limb ischemia in db/db mice, and then treated them with PACs collected from syngeneic, healthy animals or with conditioned media harvested from such cells. It turned out that a single intramuscular injection of conditioned media facilitates restoration of perfusion. Importantly, delivery of PACs themselves was ineffective, or even detrimental at early time points, what might suggest that a massive death of injected cells could induce local inflammatory reaction. The most possible explanation for lack of efficacy of PAC delivery can be a low proportion of cells surviving at the site of transplantation. Recently we showed that after intradermal PAC injection approximately 70% of cells were removed in first six hours, and less than 1% were present one week later at the site of delivery [12]. This supposition on too low number of surviving cells can be indirectly supported by results described by Di Santo and colleagues, who showed beneficial, proangiogenic effects of both conditioned media and cells in a rat model of chronic hind limb ischemia. They used, however, serial intramuscular injections (instead of single injection) and higher number of cells (1 × 10^6^ instead of 2.5 × 10^5^ cells) [63]. Our results indicate that cell free conditioned media may be an alternative for therapeutic angiogenesis in ischemic muscles. Beneficial effects of conditioned media from PACs, adipose-derived or bone marrow-derived mesenchymal stem cells have already been described by our and other teams in ischemic muscles in mice and rats [12,64,65].

Analysis of gene expression showed that PACs isolated from db/db mice displayed higher VEGFR-2 and lower CXCR4 receptor levels than their wild type counterparts. Incubation of PACs with rosiglitazone did not affect VEGFR-2 and CXCR4 expressions, but upregulated VEGF and KC, both in wild type and db/db PACs. These results are in accordance to earlier studies. Transcriptional upregulation of VEGF was first time reported in rat and human vascular smooth muscle cells stimulated *in vitro* with ciglitazone or rosiglitazone [24,25]. These observations were then confirmed in vascular smooth muscle cells in human atheromatous aortas [66,67]. Moreover, PPARγ activation increased VEGF production in murine adipose tissue [23,68] and cornea [69]. Also in patients with type-2 diabetes the level of VEGF and IL-8 were increased under pioglitazone therapy [70]. Importantly, administration of PPARγ agonists improved revascularization in muscle subjected to hind limb ischemia in mice [71] and enhanced angiogenesis in ischemic brain in rats [32]. Additionally, we noticed that rosiglitazone increased expression of PRG4 and angiotensinogen in cultured PACs. This observation seems interesting as PRG4 can promote development of hemangioblasts [72] and similar role has been ascribed to the angiotensin converting enzyme [73].

Influence of diabetes on PPARγ expression is not apparent. Direct comparison showed the same levels of PPARγ in adipocytes and aorta of db/db (diabetic) and db/m (non-diabetic) mice [74]. Analysis performed in rats, where diabetes was induced with streptozotocin (STZ), suggested the tissue specific effects – expression of PPARγ was increased in aorta, but decreased in renal cortex or retina [75]. However, decreased expression of PPARγ can also be expected in diabetic and obese patients, as inverse correlation was found between the body mass index (BMI) and PPARγ level in human adipocytes [76]. Noteworthy, PPARγ is downregulated by AGE [77] and TNFα [78], overproduced in obesity and type 2 diabetes. Recently, Biscetti et al. described the reduced expression and activity of PPARγ in muscles of STZ-treated diabetic mice subjected to hind limb ischemia. This was accompanied by decreased production of VEGF and impaired neovascularization [69]. In accordance, we found significantly lower level of PPARγ mRNA either in bone marrow or in cultured PACs derived from db/db mice. This decrease had possibly a functional effect, as suggested by changes in expression of PPARγ target genes.

One can than suppose that tight regulation of PPARγ expression is necessary for proper angiogenesis. Hence, PPARγ^−/−^ mice displayed vasculature defects in placentas, correlated with an unsettled balance of pro- and antiangiogenic factors [30,45]. On the other hand, treatment of pregnant wild-type mice with rosiglitazone also resulted in a disorganization of the placental microvasculature [30]. Our results indicate, however, that ~50% decrease in expression of PPARγ in normoglycemic PPARγ^+/−^ mice does not affect angiogenesis in wound healing or hind limb ischemia models. What is more, PACs isolated from PPARγ^+/−^ mice do not resemble gene expression pattern observed in diabetic cells. Thus, we assume that similar decrease found in diabetic animals does not contribute to impaired angiogenesis.

In summary, activation of PPARγ with rosiglitazone improves angiogenic potential of PACs and endothelial cells, impaired in diabetes. We showed that expression of PPARγ is decreased in diabetic db/db mice, however similar decrease in normoglycemic PPARγ^+/−^ mice does not affect angiogenesis. This indicate that impaired angiogenic potential in db/db mice is independent of decreased expression of PPARγ. We showed also that paracrine activity of PACs is sufficient to improve neovascularization in ischemic muscle of diabetic mice. Therefore, application of conditioned media harvested from PACs can be proposed as effective and safe alternative to proangiogenic cell transplantation.

## Additional files

Additional file 1
**Supplemental Methods and Results.**
Additional file 2: Figure S1 Characterization of PACs isolated from murine bone marrow and cultured *in vitro* for 10 days. **A**: Bright field photograph of PACs. Representative picture, magnification ×100. **B**: Uptake of DiI-AcLDL (red) by PACs. Representative picture, magnification ×100. **C**: Binding of BS1 lectin (green) by PACs. Representative picture, magnification ×100. **D**: Formation of cords by PACs stained with DiI-AcLDL (red) and seeded on matrigel. Representative picture, magnification ×100. **E**: Fraction of cells expressing CD45, KDR, CXCR4 or SCA-1 antigens in PAC populations isolated from wild type (WT) or diabetic (db/db) mice after a 10-day incubation period. Flow cytometry phenotyping. Each bar represents mean + SEM. N = 3, ***p* < 0.01, ****p* < 0.001 *versus* WT. Additional file 3: Figure S2 Body mass and blood biochemical parameters in wild type (WT) and diabetic (db/db) mice fed daily for two weeks either with vehicle (WT and db/db) or with rosiglitazone (db/db + ROSI, 10 mg/kg of body weight) and analyzed 14 days after surgery. **A**: Body mass before treatment (d 0) and at 14^th^ day of treatment (d 14). **B**: Concentration of glucose. **C**: Percentage of glycated hemoglobin. **D**: Concentration of cholesterol. **E**: Concentration of triglycerides. Automated biochemistry analyzer. Each bar represents mean + SEM. N = 20, ****p* < 0.001 *versus* WT, ^##^ *p* < 0.01, ^###^ *p* < 0.001 *versus* untreated db/db, ^^^^^p < 0.001 *versus* d 0). Additional file 4: Figure S3 Expression of angiogenic genes in cultured PACs and presence of CD45^−^KDR^+^Sca-1^+^ cells in bone marrow or peripheral blood of wild type (WT) and PPARγ haplodeficient (PPARγ^+/−^) mice. **A**: VEGF in PACs. **B**: KDR in PACs. **C**: SDF-1 in PACs. **D**: CXCR4 in PACs. Quantitative RT-PCR. EF2 serves as an internal control. **E**: Percentage of CD45^−^KDR^+^Sca-1^+^ cells in bone marrow. **F**: Percentage of CD45^−^KDR^+^Sca-1^+^ cells in blood. Multicolor FACS phenotyping. Each bar represents mean + SEM. N = 5-6 (Figure S3 A-D), N = 5-9 (Figure S3 E-F). Additional file 5: Figure S4 Expression of angiogenic genes in bone marrow and gastrocnemius muscle of wild type (WT) or PPARγ haplodeficient (PPARγ+/−) mice, untreated (control) or one day after hind limb ischemia (HLI). **A**: VEGF in bone marrow. **B**: VEGF in muscle. **C**: SDF-1 in bone marrow. **D**: SDF-1 in muscle. **E**: KDR in bone marrow. **F**: KDR in muscle. **G**: CXCR4 in bone marrow. **H**: CXCR4 in muscle. Quantitative RT-PCR. EF2 serves as an internal control. Each bar represents mean + SEM. N = 5-6, **p* < 0.05, ***p* < 0.01, ****p* < 0.001 *versus* control.
