# Erratum: PPARγ activation but not PPARγ haplodeficiency affects proangiogenic potential of endothelial cells and bone marrow-derived progenitors

**DOI:** 10.1186/s12933-015-0205-4

**Published:** 2015-05-29

**Authors:** Jerzy Kotlinowski, Anna Grochot-Przeczek, Hevidar Taha, Magdalena Kozakowska, Bartosz Pilecki, Klaudia Skrzypek, Jakub Zimoch, Aleksandra Bartelik, Rafal Derlacz, Anton J G Horrevoets, Attila Pap, Laszlo Nagy, Jozef Dulak, Alicja Jozkowicz

**Affiliations:** Department of Medical Biotechnology, Faculty of Biochemistry, Biophysics and Biotechnology, Jagiellonian University, Gronostajowa 7, 30-387 Krakow, Poland; Department of Biophysics, Faculty of Biochemistry, Biophysics and Biotechnology, Jagiellonian University, Krakow, Poland; R&D Department, Adamed Ltd, Pienkow, Poland; Department of Metabolic Regulation, Institute of Biochemistry, Faculty of Biology, University of Warsaw, Warsaw, Poland; Department of Molecular Cell Biology and Immunology, VU University Medical Center, Amsterdam, The Netherlands; Department of Biochemistry and Molecular Biology, Faculty of Medicine, University of Debrecen, Debrecen, Hungary; MTA-DE “Lendület” Immunogenomics Research Group, University of Debrecen, Debrecen, Hungary; Current address: Sanford-Burnham Medical Research Institute, Orlando, FL USA; Current affiliation: Department of Molecular Neuropharmacology, Institute of Pharmacology Polish Academy of Science, Krakow, Poland

## Erratum

After publication of this work [1], we noted that we inadvertently and accidentally failed to include one coauthor. The full list of authors has now been added and the Authors' contributions and Competing interests section modified accordingly.
