# Supplementary material for: PPARγ activation but not PPARγ haplodeficiency affects proangiogenic potential of endothelial cells and bone marrow-derived progenitors
Source: Cardiovasc Diabetol. 2014 Nov 1;13:150. doi: 10.1186/s12933-014-0150-7 (PMC4233236; doi:10.1186/s12933-014-0150-7)
Supplement: Additional file 1 — Supplemental Methods and Results. [file 12933_2014_150_MOESM1_ESM.docx]

**Supplemental Methods and Results**

**Microarrays.** Transcriptome analysis was performed by the company ServiceXS Ltd. (Leiden, Netherlands) using Illumina MouseWG-6 microarrays, according to manufacturer's instruction. Briefly, total RNA was isolated from the confluent PACs incubated for 24 h in normoxia (21% O_2_) or hypoxia (2% O_2_). Quality of isolated RNA was checked using Agilent 2100 Bioanalyzer. Samples with RIN (RNA integrity number) ≥ 9 were used for microarray analysis, according to vendor's protocols. Aliquots (500 ng) of RNA were used for the synthesis of biotinylated complementary RNA (cRNA). Next, cRNA (1500 ng) was used for hybridization. After washing steps, the detection of positives with streptavidin-cyanine-3 conjugate was performed. Signal intensities were measured using confocal scanner iScan (Illumina), whereas Beadstudio v3 module was employed for data analysis. After normalization the raw data were further subjected to gene set enrichment analysis with GeneGo and Metacore software.

**Tube formation on Matrigel.** To assess the paracrine effects of PACs, the conditioned media were prepared by incubation of confluent cells in EBM-2 medium containing 2% FBS for 24 h. Next, their aliquots (200 μL/well) were used for stimulation of HUVECs seeded on the growth factor-reduced Matrigel (50 μL). Empty medium (EBM-2 with 2% FBS) and complete medium (EGM-2MV containing 10% FBS and 30 ng/mL of VEGF) were applied as a negative and positive control, respectively. Endothelial tube formation was evaluated after incubation of cells for 16 h at 37°C using Nikon Eclipse TX-100 microscope.

**Outgrowth of capillaries from PAC spheroid.** Spheroids were formed from 600 PACs seeded for 48 h into U-shaped non-adherent 96-well plates in EGM-2MV complete medium supplemented with methylcellulose (12 g/L). Next, spheres were collected, spun down and suspended in 30% FBS in methylcellulose. Such suspension was then mixed with collagen, plated into 24-well plate and, after collagen gelatinization, covered with EGM-2MV medium (200 μL/well). Plates were incubated in standard conditions for 48 h before the capillary outgrowth measurements. Microscopic analysis was done using Nikon Eclipse TX-100 microscope.

**Aortic ring assay.** Murine aorta was excised, connective tissue was removed and small rings were cut and placed on Matrigel on 96-well plate. Rings were seeded in EGM2 medium containing 2% FBS and stimulated next day with rosiglitazone (10 μmol/L) and/or GW9662 (10 μmol/L). PPARγ inhibitor was added 30 minutes before rosiglitazone. Growth of new capillaries was monitored and numbers of sprouting vessels were analyzed at day 5 using Nikon Eclipse TX-100 microscope.

**Proliferation assay**. PACs were seeded in chamber slides and cultured in standard conditions until reaching a confluence of 70%. Then culture medium was changed to EGM-2 with 2% FBS for 24 h and the cells were stimulated with rosiglitazone (10 µmol/L) and/or GW9662 (10 μmol/L, added 30 minutes before rosiglitazone) for next 24 h. In the next step the cells were washed with PBS, fixed with paraformaldehyde and ethanol, and then permeabilized with 0.2% Triton-X100. Proliferating cells were stained with anti-mouse PCNA antibody overnight at 4°C. Secondary antibody was applied for 1 h at room temperature, whereas cell nuclei were stained with DAPI. PCNA-positive cells were counted using the fluorescence microscope (Nikon Eclipse TX-100)

**PPRE-luciferase reporter assay.** In vitro cultured cells were transfected wtih reporter plasmid encoding luciferase under control of PPRE. Next day, cells were stimulated with rosiglitazone (10 μmol/L) and luciferase activity was measured [1].

**Milliplex®MAP.** Concentrations of cytokines in plasma were analyzed using Luminex technology in 96-well plates according to vendor's protocol. Briefly, samples were mixed with buffer containing the standards and microspheres and incubated for 2 h. Next, after buffer aspiration and washing with PBS, the secondary antibodies conjugated with biotin were added. Prior to fluorescence measurement the samples were supplemented with a streptavidin-phycoerythrin complex.

**Blood morphology and biochemistry.** Blood samples for hematological analysis were collected into EDTA/K coated tubes and stored on ice. They were mixed just before measurement with Scil Vet ABC buffer (Horiba) and analyzed using ABC Vet Animal Blood Counter (Horiba). For biochemical tests, venous blood was collected into EDTA coated tubes, incubated for 1 h at room temperature, and then centrifuged (10 minutes, 9000 *g*, 4ºC). Serum was collected into a new tube and stored at -80ºC till further analysis. Measurements of glucose, cholesterol, triglycerides, albumin and HbA1c contents were done using the biochemical analyzer ABX Pentra 400 (Horiba), according to manufacturer's instructions.

**Leukocyte infiltration.** Frozen sections of muscles (6 μm) were stained with hematoxylin/eosin according to standard protocols. The assessment of leukocyte infiltration levels was based on arbitrary scale from 0 (no infiltration) to 3 (massive infiltration) using Nikon Eclipse TX-100 microscope.

**Supplementary Table S1.** Sequences of primers used in qRT-PCR analysis of gene expression. Ta – annealing temperature. K – G or T.

| **gene** | **forward 5'–3'** | **reverse 5'–3'** | **Ta** | **length of product (bp)** |
| --- | --- | --- | --- | --- |
| **VEGF** | ATG CGG ATC AAA CCT CAC CAA GGC | TTA ACT CAA GCT GCC TCG CCT TGC | 60 | 220 |
| **VEGFR-1** | GCA CCT ATG CST GCA GAG C | TCT TTC AAT AAA CAG CGT GCT G | 58 | 266 |
| **VEGFR-2** | CCT CAC CTG TTT CCT GTA TGG AG | GAK GCC ACA GAC TCC CTG C | 60 | 300 |
| **SDF-1** | CCT TCA GAT TGT TGC ACG GCT GA | CCC ACC ACT GCC CTT GCA TC | 60 | 181 |
| **CXCR4** | AAA CCT CTG AGG CGT TTG GT | AGC AGG GTT CCT TGT TGG AG | 60 | 149 |
| **CXCR7** | CTG AGG TCA CTT GGT CGC TC | TGC ACA GTG TCC ACC ACA AT | 60 | 129 |
| **PRG4** | CGT TGC ATC CGA GAA CCA TG | CAT CTC CCT GCA CAG CTT GA | 62 | 118 |
| **AGT** | CCA CTG GAG GGG GTC AGT ACA | GAG ATG CTG TTG TCC ACC CAG A | 62 | 125 |
| **PPARγ** | GTG ATC TTA ACT GCC GGA TCC AC | CTC TGG GTT CAG CTG GTC GAT A | 60 | 179 |
| **EF2** | GCG GTC AGC ACA ATG GCA TA | GCG GTC AGC ACA ATG GCA TA | 60 | 210 |
| **MMP2** | ACA CTG GGA CCT GTC ACT CC | GCG AAG AAC ACA GCC TTC TC | 58 | 230 |
| **MMP9** | TGT GGA TGT TTT TGA TGC TAT TG | CGG AGT CCA GCG TTG CA | 60 | 158 |
| **NOX4** | ACA TTC ACC AAA TGT TGG GCC T | CCT GCT AGG GAC CTT CTG TGA | 60 | 143 |
| **p22phox** | GTC ATG GGG CAG ATC GAG TG | GTC ATG GGG CAG ATC GAG TG | 60 | 147 |
| **GST-III** | GCT CCA CCT AGC CAT CAA CG | CCT CCA ACG TGT TCT GGT GG | 58 | 134 |
| **TNFR-1** | AGG ACT CAG GTA CTG CGG TG | AGT AGA CTT CGG GCC TCC AC | 60 | 117 |
| **Selplg** | GAC AGA ATG ACC TCG CGC TG | GGA CAA TGG TCT AAG CGC CC | 60 | 135 |
| **NOS3** | TTG GTG TTT GGC TGC CGA TGC | GGT GAA CCT CCG CGG CTA GC | 60 | 175 |
| **CAV1** | CCA AGC ATC TCA ACG ACG  AC | GGC AAA GTA AAT GCC CCA  GA | 60 | 194 |

**Supplementary Table S2.** Hematological parameters in wild type (WT) or diabetic (db/db) mice fed daily for two weeks either with vehicle (WT and db/db) or with rosiglitazone (db/db+ROSI, 10 mg/kg of body weight) and analyzed at day 14^th^. WBC – white blood cells, RBC – red blood cells, HGB – hemoglobin, HCT – hematocrit, PLT – platelets, MCV – mean corpuscular volume, MCH – mean corpuscular hemoglobin, MCHC – mean corpuscular hemoglobin concentration. Means + SEM. N=8-10 individuals for each parameter, **^**^** *p*<0.01, **^***^** *p*<0.001 *versus* WT.

| **Parameter** | **WT** | **db/db** | **db/db+ROSI** |
| --- | --- | --- | --- |
| **WBC** [10^3^/mm^3^] | 12.3 ± 1.3 | 6.8 ± 0.8 ** | 5.5 ± 0.4 *** |
| **RBC** [10^6^/mm^3^] | 10.2 ± 0.5 | 11.0 ± 0.5 | 10.3 ± 0.1 |
| **HGB** [g/dL] | 16.0 ± 0.8 | 18.0 ± 0.8 | 16.5 ± 0.2 |
| **HCT** [%] | 45.3 ± 2.3 | 49.4 ± 0.6 | 49.2 ± 0.7 |
| **PLT** [10^3^/mm^3^] | 1084.2 ± 107.4 | 1363.6 ± 123.7 | 1126.7 ± 107.9 |
| **MCV** [μm^3^] | 44.1 ± 0.1 | 47.5 ± 0.4 | 47.8 ± 0.1 |
| **MCH** [pg/cell] | 15.7 ± 0.1 | 16.4 ± 0.2 | 16.1 ± 0.1 |
| **MCHC** [g/dL] | 35.4 ± 0.1 | 34.6 ± 0.3 | 33.6 ± 0.2 |

1. Funovics P, Brostjan C, Nigisch A, Fila A, Grochot A, Mleczko K, Was H, Weigel G, Dulak J, Jozkowicz A: **Effects of 15d-PGJ_2_ on VEGF-induced angiogenic activities and expression of VEGF receptors in endothelial cells.** *Prostaglandin Other Lipid Mediat* 2006, **79**:230-244.
