# Supplementary figures and images for: PPARγ activation but not PPARγ haplodeficiency affects proangiogenic potential of endothelial cells and bone marrow-derived progenitors
Source: Cardiovasc Diabetol. 2014 Nov 1;13:150. doi: 10.1186/s12933-014-0150-7 (PMC4233236; doi:10.1186/s12933-014-0150-7)

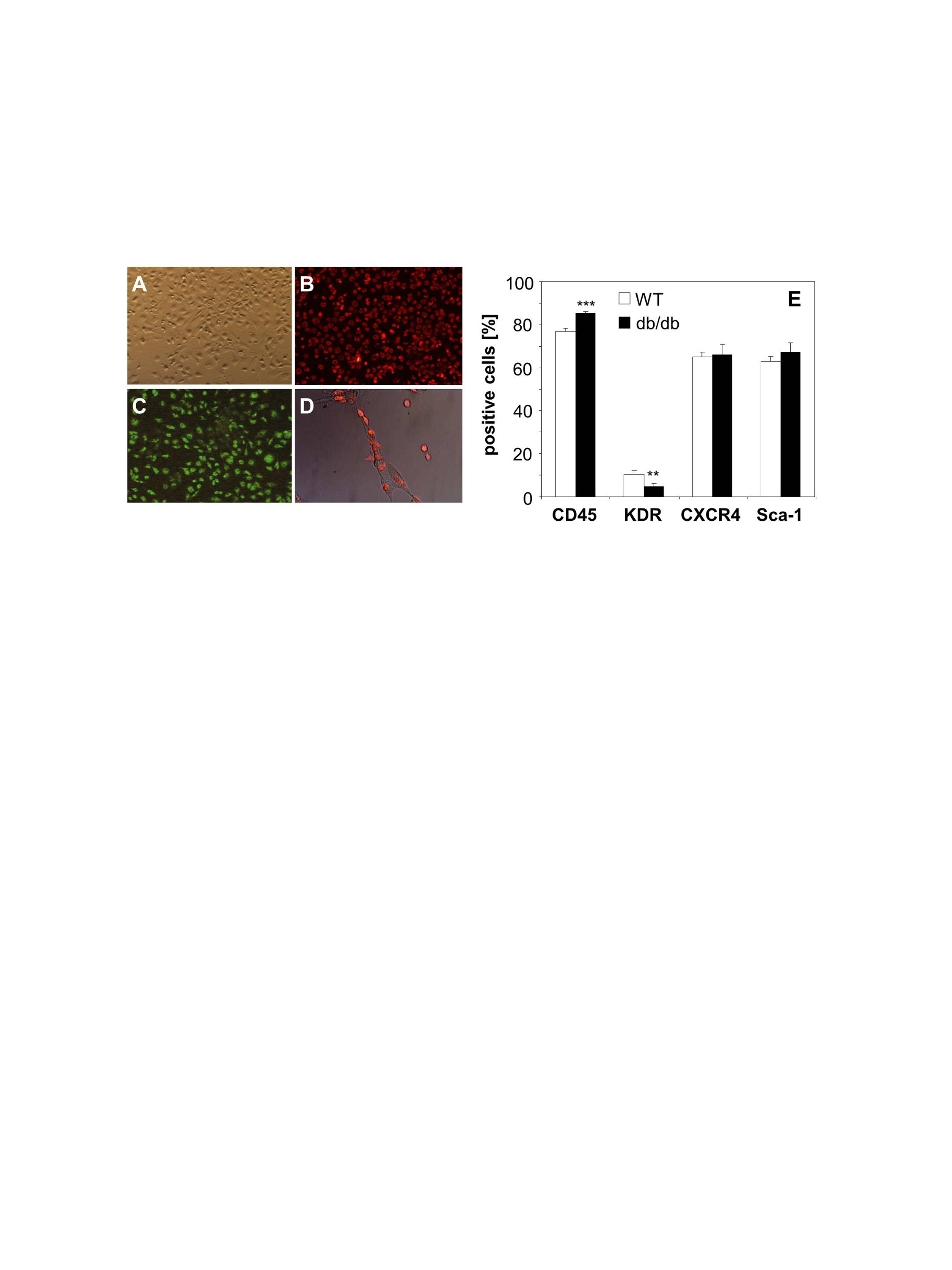

Supplement: Additional file 2: Figure S1 — Characterization of PACs isolated from murine bone marrow and cultured in vitro for 10 days. A: Bright field photograph of PACs. Representative picture, magnification ×100. B: Uptake of DiI-AcLDL (red) by PACs. Representative picture, magnification ×100. C: Binding of BS1 lectin (green) by PACs. Representative picture, magnification ×100. D: Formation of cords by PACs stained with DiI-AcLDL (red) and seeded on matrigel. Representative picture, magnification ×100. E: Fraction of cells expressing CD45, KDR, CXCR4 or SCA-1 antigens in PAC populations isolated from wild type (WT) or diabetic (db/db) mice after a 10-day incubation period. Flow cytometry phenotyping. Each bar represents mean + SEM. N = 3, **p < 0.01, ***p < 0.001 versus WT. [file 12933_2014_150_MOESM2_ESM.tiff]

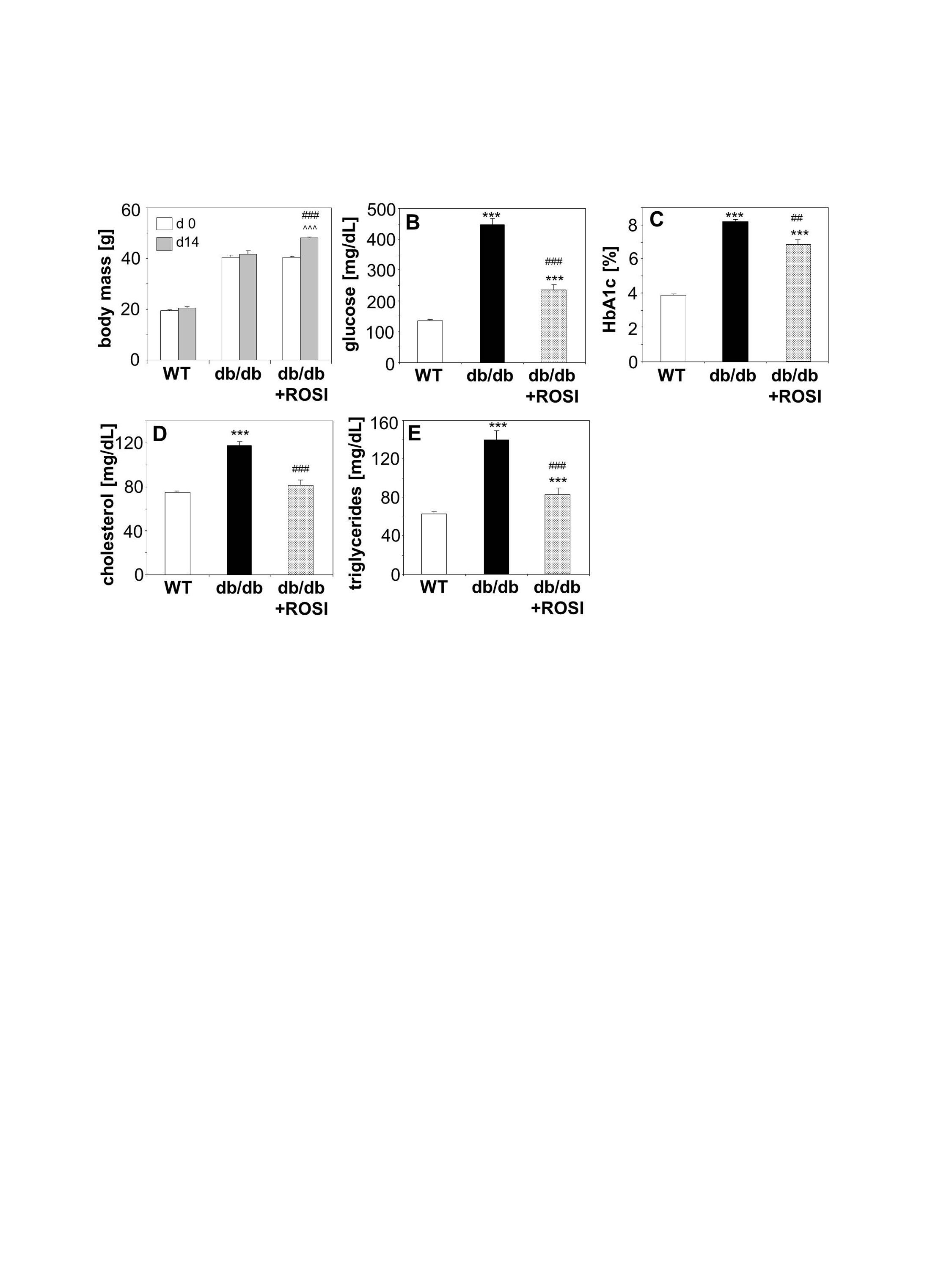

Supplement: Additional file 3: Figure S2 — Body mass and blood biochemical parameters in wild type (WT) and diabetic (db/db) mice fed daily for two weeks either with vehicle (WT and db/db) or with rosiglitazone (db/db + ROSI, 10 mg/kg of body weight) and analyzed 14 days after surgery. A: Body mass before treatment (d 0) and at 14th day of treatment (d 14). B: Concentration of glucose. C: Percentage of glycated hemoglobin. D: Concentration of cholesterol. E: Concentration of triglycerides. Automated biochemistry analyzer. Each bar represents mean + SEM. N = 20, ***p < 0.001 versus WT, ## p < 0.01, ### p < 0.001 versus untreated db/db, ^^^p < 0.001 versus d 0). [file 12933_2014_150_MOESM3_ESM.tiff]

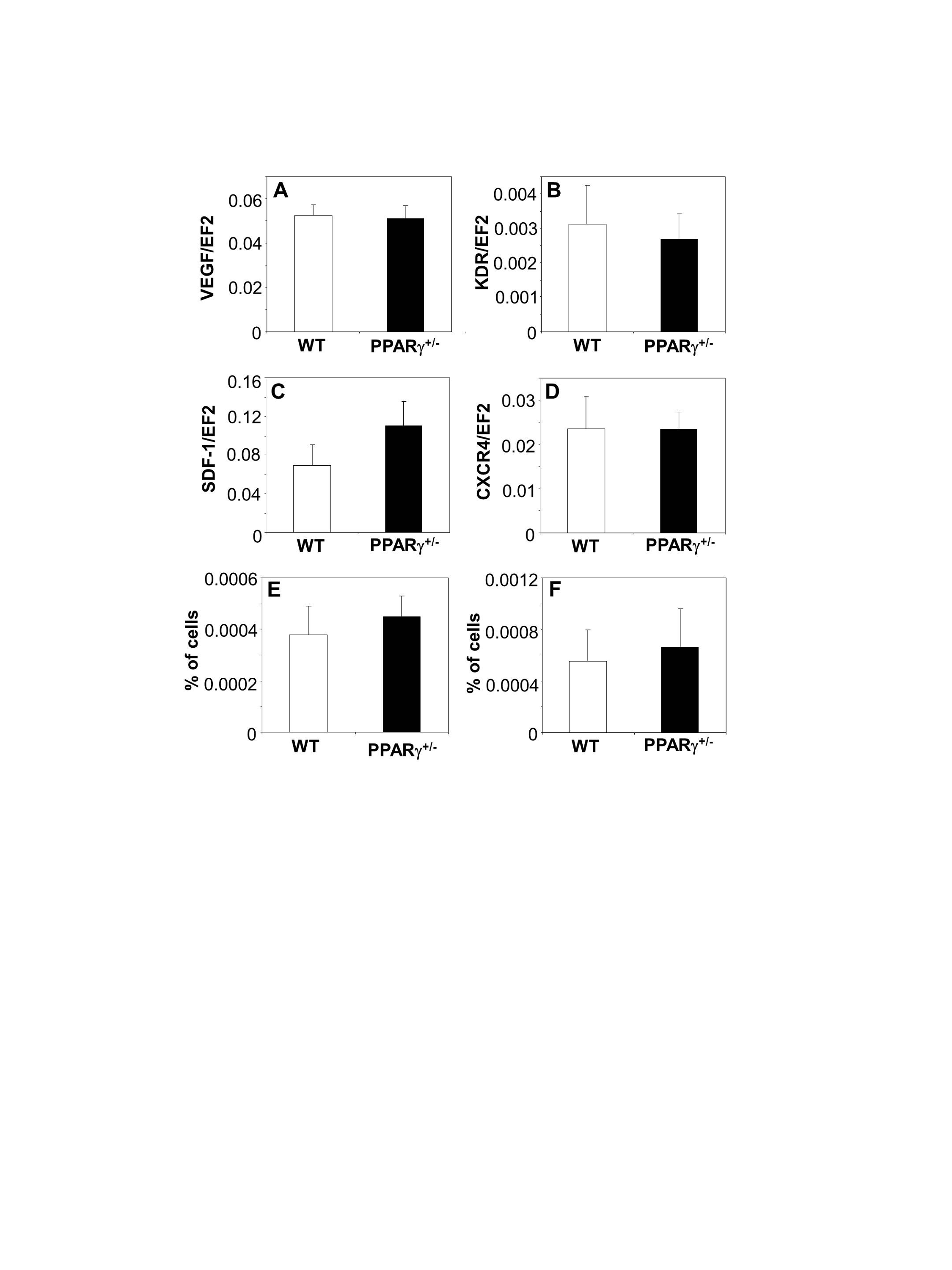

Supplement: Additional file 4: Figure S3 — Expression of angiogenic genes in cultured PACs and presence of CD45−KDR+Sca-1+ cells in bone marrow or peripheral blood of wild type (WT) and PPARγ haplodeficient (PPARγ+/−) mice. A: VEGF in PACs. B: KDR in PACs. C: SDF-1 in PACs. D: CXCR4 in PACs. Quantitative RT-PCR. EF2 serves as an internal control. E: Percentage of CD45−KDR+Sca-1+ cells in bone marrow. F: Percentage of CD45−KDR+Sca-1+ cells in blood. Multicolor FACS phenotyping. Each bar represents mean + SEM. N = 5-6 (Figure S3 A-D), N = 5-9 (Figure S3 E-F). [file 12933_2014_150_MOESM4_ESM.tiff]

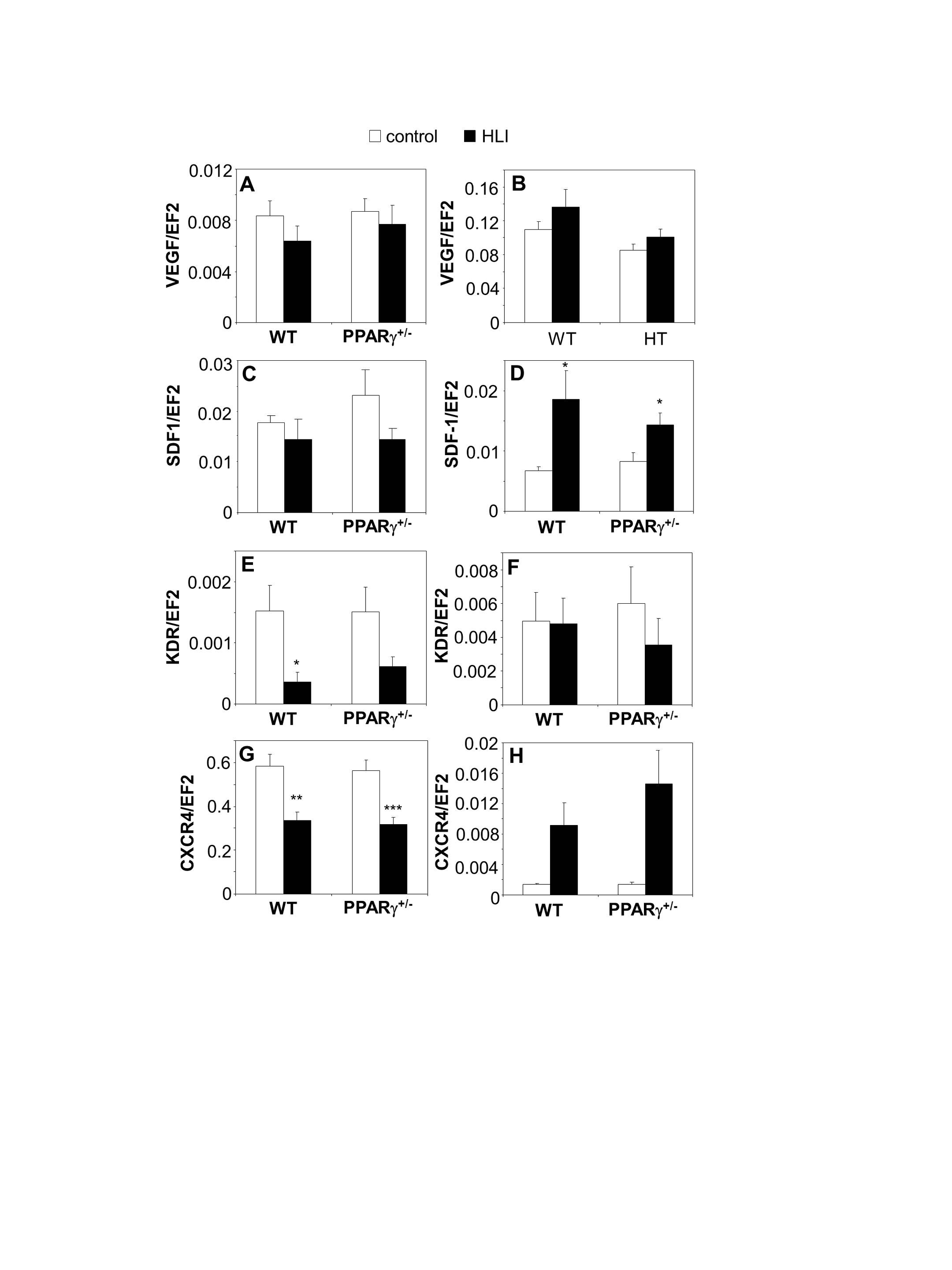

Supplement: Additional file 5: Figure S4 — Expression of angiogenic genes in bone marrow and gastrocnemius muscle of wild type (WT) or PPARγ haplodeficient (PPARγ+/−) mice, untreated (control) or one day after hind limb ischemia (HLI). A: VEGF in bone marrow. B: VEGF in muscle. C: SDF-1 in bone marrow. D: SDF-1 in muscle. E: KDR in bone marrow. F: KDR in muscle. G: CXCR4 in bone marrow. H: CXCR4 in muscle. Quantitative RT-PCR. EF2 serves as an internal control. Each bar represents mean + SEM. N = 5-6, *p < 0.05, **p < 0.01, ***p < 0.001 versus control. [file 12933_2014_150_MOESM5_ESM.tiff]
